# Novel CH25H^+^ and OASL^+^ microglia subclusters play distinct roles in cerebral ischemic stroke

**DOI:** 10.1186/s12974-023-02799-6

**Published:** 2023-05-15

**Authors:** Yueman Zhang, Yunlu Guo, Ruqi Li, Tingting Huang, Yan Li, Wanqin Xie, Chen Chen, Weijie Chen, Jieqing Wan, Weifeng Yu, Peiying Li

**Affiliations:** 1 Department of Anesthesiology, Key Laboratory of the Ministry of Education, Renji Hospital, Shanghai Jiao Tong University School of Medicine, 160 Pujian Road, Shanghai, 200127 China; 2 Department of Neurological Surgery, Renji Hospital, Shanghai Jiao Tong University School of Medicine, Shanghai, China; 3 Clinical Research Center, Renji Hospital, Shanghai Jiao Tong University School of Medicine, 160 Pujian Road, Shanghai, 200127 China

**Keywords:** Microglia, Ischemic stroke, Single-cell RNA sequencing, Microglia heterogeneity, Neuroprotection

## Abstract

**Background:**

Microglial polarization is one of the most promising therapeutic targets for multiple central nervous system (CNS) disorders, including ischemic stroke. However, detailed transcriptional alteration of microglia following cerebral ischemic stroke remains largely unclear.

**Methods:**

Focal cerebral ischemia was induced by transient middle cerebral artery occlusion (tMCAO) for 60 min in mice. Single-cell RNA sequencing (scRNA-seq) was performed using ischemic brain tissues from tMCAO and sham mice 3 days after surgery. *Ch25h*^−/−^ mice were used to investigate the role of specific microglia subcluster on post-stroke infarct volume and neuroinflammation.

**Results:**

We identified a relatively homeostatic subcluster with enhanced antigen processing and three “ischemic stroke associated microglia” (ISAM): MKI67^+^, CH25H^+^ and OASL^+^ subclusters. We found the MKI67^+^ subcluster undergo proliferation and differentiation into CH25H^+^ and OASL^+^ subclusters. CH25H^+^ microglia was a critical subcluster of ISAM that exhibited increased phagocytosis and neuroprotective property after stroke. *Ch25h*^−/−^ mice developed significantly increased infarct volume following ischemic stroke compared to* Ch25h*^+/−^. Meanwhile, the OASL^+^ subcluster accumulated in the ischemic brain and was associated with the evolving of neuroinflammation after stroke, which was further aggravated in the aged mice brain.

**Conclusions:**

Our data reveal previously unrecognized roles of the newly defined CH25H^+^ and OASL^+^ microglia subclusters following ischemic stroke, with novel insights for precise microglia modulation towards stroke therapy.

**Supplementary Information:**

The online version contains supplementary material available at 10.1186/s12974-023-02799-6.

## Introduction

Stroke is a leading cause of permanent disability and death with limited treatment options [1]. The inflammatory response in the brain occurring hours to days after stroke is associated with the complex pathology of brain injury after stroke [2, 3]. Microglia, as part of the innate immune system, are considered as orchestrators of neuroinflammation in different neurological disorders, such as acute brain injuries and neurodegenerative diseases [4, 5]. In response to acute brain injury, activated microglia develop into either classically activated (M1) or alternatively activated (M2) phenotypes [4]. Accumulating evidence suggests that the transition from proinflammatory M1 microglia to anti-inflammatory M2 microglia is a promising therapeutic approach to treat ischemic stroke patients [6, 7]. Targeting microglial polarization provides a new avenue to the ischemic stroke.

Progress in single-cell transcriptomic techniques has facilitated the understanding of microglia heterogeneity and revealed multiple disease-specific microglia subclusters [8, 9], including disease-associated microglia (DAM) in Alzheimer’s disease (AD) [10], lipid-droplet-accumulating microglia (LDAM) in aging [11], oxidized phosphatidylcholines (OxPC) clearing associated microglia in MS [12, 13], Fn1^+^ microglia in spinal cord injury [14], and Grn^+^ microglia in TDP-43 proteinopathy [15]. The above microglia subclusters display critical roles in regulating a plethora of disease progression, for example, LDAM exhibits lysosome dysfunction and promote neuroinflammation [11], while Fn1^+^ microglia organize scar-free spinal cord repair [14]. Recent single-cell analysis have revealed the cellular heterogeneity and the transcriptional changes of microglia during the evolving of neuroinflammation following cerebral ischemic stroke. For instance, Zheng et al. mapped the cell populations in the mouse model of tMCAO and identified diverse differentiation of five distinct subtypes of microglia after ischemic stroke [16]. Guo et al. revealed the impact of ischemic stroke on specific genes and pathways of different cell types [17]. However, the regulatory mechanism of microglia subclusters following cerebral ischemic stroke remains largely unclear. Therefore, there’s an urgent need to identify specific microglia subclusters that could serve as therapeutic targets for stroke therapy.

In this study, we identified 3 distinct ischemic stroke-associated microglia (ISAM) specifically marked with MKI67 (MG4), OASL (MG5), and CH25H (MG6), respectively. We identified CH25H^+^ microglia as a neuroprotective subpopulation, and found *Ch25h* deficiency in microglia exacerbated ischemic brain injury and neuroinflammation after stroke. We also identified OASL^+^ subcluster with upregulated interferon-response-related gene, which is associated with age-dependent deteriorated infarct growth after ischemic stroke. Collectively, our findings identified MKI67^+^ microglia as the proliferative, CH25H^+^ as the neuroprotective, while OASL^+^ as the proinflammatory microglia subcluster which was associated with ischemic brain injury 3 days after stroke. These findings could pave the way for developing more precise microglia modulation in the pursuit of better stroke therapy.

## Methods

### Animals

Male C57/BL6 and *Ch25h*^*−/−*^ mice were acquired from Shanghai SLAC Laboratory Animals and the GemPharmatech Co. Ltd., which maintained in accordance with conventional laboratory settings (22 °C, a 12-h light–dark cycle, and free access to food and water). All studies were conducted in line with the Institutional Guide for the Care and Use of Laboratory Animals and were approved by Renji Hospital Institutional Animal Care and Use Committee.

### Cerebral ischemia model

Mice were anesthetized with 3% isoflurane in 67%:30% N_2_O/O_2_ (induction), until they were unresponsive to the tail pinch test and were then fitted with a nose cone providing 1.5% isoflurane for anesthesia maintenance. We used two different models to induce cerebral ischemia: transient middle cerebral artery occlusion (tMCAO) and distal middle cerebral artery occlusion (dMCAO). We used tMCAO to induce focal cerebral ischemia in the mice for 60 min, and dMCAO to induce permanent ischemia in the experiments of the aged mice, details and study timeline can be found in Additional file 2.

### CBF measurement

The success of tMCAO/dMCAO model induction was monitored by the laser speckle technique. Briefly, cerebral perfusion images were acquired using the PeriCam PSI System (Perimed) positioned above the mice head before, during ischemia, and after reperfusion. Speckle contrast was defined as the ratio of SD of pixel intensity to the mean pixel intensity. The speckle visibility relative to the velocity of the light-scattering particles was converted to correlation time values, which are inversely and linearly proportional to the mean blood velocity. CBF changes were expressed as the percentage of pre- tMCAO/dMCAO baselines.Animals were excluded if the suture was not successfully placed to occlude the tMCAO which was monitored by two-dimensional laser speckle. Animal data reporting followed The ARRIVE 2.0 guidelines [18] and STAIR X Stroke recommendations [19].

### Immunofluorescence

We perfused the euthanized mice with ice-cold phosphate-buffered saline (PBS) followed by 4% paraformaldehyde (PFA) through the left ventricle to fix the brain tissue. The intactly extracted brain tissues were post-fixed in 4% PFA for 24 h and dehydrated in 30% sucrose solution for 48 h at 4 °C. We then embedded the brains in OCT and cryosectioned at a thickness of 25 μm. Coronal brain sections were incubated with 10% normal donkey serum for 30 min at room temperature in PBS containing 0.1% Triton X-100 followed by incubation with appropriate primary antibodies overnight at 4 °C in the same buffer. The anti-Iba-1 (1:500, catalog: ab178846, Abcam, Britain), anti-Mki67 (1:500, catalog: 14-5698-821, eBioscience™, USA), anti-OASL (1:300, catalog: ab191701, Abcam, Britain), anti-CH25H (1:300, catalog: H00009023, Novus Biologicals, USA), anti-iNOS (1:300, BD Biosciences), and anti-CD68 (1:500, catalog: ab53444, Abcam, Britain) primary antibodies were used. After primary antibody incubation, sections were washed four times at room temperature, followed by incubation with appropriate fluorescent-labeled secondary antibodies (1:1000) for 1 h at room temperature. We verified the specificity of antibodies by testing them on brain tissue sections from sham-operated mice. We also performed secondary antibody-only control staining to exclude non-specific binding. All the confocal images were captured on laser scanning confocal microscope (Olympus Fluoview FV3000, Olympus). The numbers of target immunopositive cells were quantified by a blinded investigator using NIH Image J (1.52a). Three randomly selected microscopic fields in the cortex on each section were analyzed for each brain by a blinded investigator. The immunopositive cells were presented as the mean percentage of cells per field.

### Single-cell dissociation

scRNA-seq experiment was performed by experimental personnel in the laboratory of NovelBio Bio-Pharm Technology Co. Ltd. The brain tissues around infarction from tMCAO and sham mice (*n* = 3/group) were surgically removed. We perfused the mice transcardially with ice-cold PBS before isolating brain tissues to obtain single-cell suspension. The tissues were kept in MACS Tissue Storage Solution (Miltenyi Biotec) until processing. The tissue samples were first washed with PBS, minced into small pieces (approximately 1mm^3^) on ice and enzymatically digested with 200μL Enzyme H and 100μL Enzyme R and 25μL Enzyme A (Adult Brain Dissociation Kit, mouse and rat, Miltenyi Biotec, 130–107–677) for 30 min at 37 °C. After digestion, samples were sieved through a 70 µm cell strainer, and centrifuged at 300 g for 5 min. After the supernatant was removed, the pelleted cells were suspended in red blood cell lysis buffer (Miltenyi Biotec) to lyse red blood cells. After washing with PBS containing 0.04% BSA, the cell pellets were re-suspended in PBS containing 0.04% BSA and re-filtered through a 35 μm cell strainer. Dissociated single cells were then stained for viability assessment using Calcein-AM (Thermo Fisher Scientific) and Draq7 (BD Biosciences).

### Single-cell RNA sequencing

BD Rhapsody system was used to capture the transcriptomic information of the brain sample-derived single cells. Single-cell capture was achieved by random distribution of a single-cell suspension across > 200,000 microwells through a limited dilution approach. Beads with oligonucleotide barcodes were added to saturation, so that a bead was paired with a cell in a microwell. The cells were lysed in the microwell to hybridize mRNA molecules to capture oligos on the beads. Beads were collected into a single tube for reverse transcription and ExoI digestion. Upon cDNA synthesis, each cDNA molecule was tagged on the 5′ end (that is, the 3′ end of a mRNA transcript) with a unique molecular identifier (UMI) and cell barcode indicating its cell of origin. Whole transcriptome libraries were prepared using the BD Rhapsody single-cell whole-transcriptome amplification (WTA) workflow including random priming and extension (RPE), RPE amplification PCR and WTA index PCR. The libraries were quantified using a High Sensitivity DNA chip (Agilent) on a Bioanalyzer 2200 and the Qubit High Sensitivity DNA assay (Thermo Fisher Scientific). Sequencing was performed by Illumina sequencer (Illumina, San Diego, CA) on a 150 bp paired-end run.

### scRNA-seq statistical analysis

We applied fastp with default parameters to filter the adaptor sequence and remove the low quality reads to obtain clean data [20]. UMI tools was applied for single-cell transcriptome analysis to identify the cell barcode whitelist [21]. The UMI-based clean data was mapped to the mouse genome (mm10 Ensemble version 91) utilizing *STAR* mapping [22] with customized parameters from the UMI-tools standard pipeline to determine the UMIs counts of each sample. To minimize the sample batch, we applied down sample analysis to the samples sequenced according to the mean reads per cell of each sample and achieved a cell expression table with a sample barcode. Cells containing over 200 expressed genes and a mitochondria UMI rate below 30% passed the cell quality filtering. Seurat R package (4.0.6) (https://satijalab.org/Seurat/) was used for cell normalization and regression based on the expression matrix according to the UMI counts of each sample and the percentage mitochondria rate to obtain the scaled data. We used the regression-based correction strategy that comes with Seurat. In addition to regressing out MT% and Library Size, we removed the batch effect from the samples. Principle component analysis (PCA) was constructed based on the scaled data with all highly variable genes. The top 10 principals were used for t-Distributed Stochastic Neighbor Embedding (tSNE) construction.

We acquired the unsupervised cell cluster result based on the top 10 principal components using graph-based cluster method (resolution = 0.8). To identify marker genes for each cluster, we used the FindAllMarkers (multiple condition comparisons) and FindMarkers (two condition comparisons) functions with Wilcoxon Rank-Sum Test under following criteria: Log FC > 0.25; *p* < 0.05; min.pct > 0.1.

We performed unsupervised clustering of all brain cells and identified 17 distinct clusters. Microglia clusters are identified and subsetted based on expression of canonical microglia gene signatures [2, 3]. Then, we further re-clustered all microglia and identified 7 microglia subclusters (MG0–MG6).

### Pseudotime analysis

We applied Single-Cell Trajectories analysis using Monocle2 (http://cole-trapnelllab.github.io/monocle-release) with DDR-Tree method and default parameters. Before Monocle analysis, we selected the marker genes of the Seurat clustering result. The raw expression counts of the cell passed filtering.

### Single-cell regulatory network inference and clustering (SCENIC) analysis

The SCENIC analysis was run on each cluster according to pySCENIC workflow, using the 20-thousand motifs database for RcisTarget and GRNboost [23] to reconstruct single-cell regulatory network and assess transcription factor regulation strength.

### Pathway enrichment analysis

Pathway enrichment analysis was used to identify the significant pathway of the differential genes according to the Gene ontology (GO) and Kyoto Encyclopedia of Genes and Genomes (KEGG) database using (clusterProfiler 3.16.1). We used Fisher’s exact test to select the significant pathway, where the threshold of significance was defined by the *p* value and false discovery rate (FDR). The cases were selected when *p* < 0.05 [24]. Specifically, for the GO analysis, we used all differentially expressed genes (DEGs) in each microglia subcluster compared to all other microglia subclusters. We used the 'FindAllMarkers' function in Seurat to identify DEGs with log fold change (logFC) threshold of 0.25 and adjusted *p* value (padj) threshold of 0.05. In addition, the ranked gene set enrichment analysis (GSEA) algorithm was used to calculate the enrichment score. The gene set variation analysis (GSVA) score of specified gene sets for each cluster was defined as the average normalize expression of the pathway-related genes. Quantitative Set Analysis for Gene Expression (QuSAGE) (2.16.1) analysis was performed to characterize the relative activation of a given gene set.

### Ligand–receptor expression and cell interactions

Cell-to-cell communication (CellChat 1.4.0) was ascertained by evaluating expression of pairs of ligands and receptors within cell populations [25]. We examined the interaction between different cell types, and gene expression of 0.2 was set as the valid cutoff point.

### Flow cytometry

For brain tissues, we homogenized the hemisphere ipsilateral to the infarct of tMCAO or sham mice using Neural Tissue Dissociation Kit (130–093-231, Miltenyi Biotec) by the gentle MACS Dissociator following the manufacturer’s instructions. The immune-cell‐enriched population was collected using Percoll gradient centrifugation. Isolated cells were stained with anti-CD11b-FITC (101,206, Biolegend), anti-CD86-BV510 (105,040, Biolegend), anti-CD45-APC-Cy7 (103,116, Biolegend), and anti-CD206-APC (141,708, Biolegend). Flow cytometry was performed on BD FACSVerse (BD Bioscience) and FlowJo software (TreeStar) was used to analyze the data.

### BV2 cell culture

The murine microglial BV2 cell line (Procell) was cultured in DMEM (Life Technologies) supplemented with 10% FBS and antibiotics (penicillin 100Uml^−1^, streptomycin 100Uml^−1^ (pen/strep), HVD Life Sciences) under standard culture conditions (95% relative humidity with 5% CO_2_ at 37 °C). Adherent cells were digested using 1 × TrypLE (Gibco).

### Bead-based phagocytosis assay

For in vitro phagocytosis assays, BV2 cells were cultured into 96-well plates. Oxygen glucose deprivation (OGD) was induced by placing the cell plate in a hypoxic chamber with a gas mixture of 1% O_2_, 5% CO_2_, and 94% N_2_ for 3 h. The bead-based phagocytosis assay was performed using fluorescently labeled latex beads (Invitrogen™ FluoSpheres™ Polystyrene Microspheres, 1.0 µm). BV2 cells were incubated with beads at a ratio of 1:10 for 2 h, then washed and fixed for analysis. Phagocytosis of beads by BV2 cells was quantified using flow cytometry and immunofluorescence.

### Behavioral tests

Sensorimotor functions was assessed by the modified Garcia Score [26], grid walk [27], and Rotarod test [28], which were performed as previously described to assess before and after surgery by investigators who were blinded to experimental group assignments. Cognitive function was analyzed using the Morris water maze test, as described previously [29]. Details can be found in Additional file 2.

### Statistical analysis

All statistics were performed using GraphPad Prism v6 or the implemented statistical tests of the respective R packages. The Shapiro–Wilk normality test was initially performed on all data sets. A two-tailed Students *t* test was used for the pairwise comparison between two groups. For behavioral tests performed at multiple timepoints on the same animals, two-way ANOVA with repeated measures was used. The rest of the data were analyzed using a one-way or two-way ANOVA as appropriate. Multiple comparison procedures were carried out to identify specific between-group differences using post hoc Tukey’s tests. Results were presented as mean ± SD. The correlation analyses were performed using Pearson correlation analysis. P ≤ 0.05 was considered statistically significant.

### Data and software availability

Raw fastq files and processed gene expression matrixes are available at the NIH GEO database (GSE210986). Partial bioinformatics analysis was used on NovelBrain Cloud Analysis Platform (www.novelbrain.com).

## Results

### Ischemic stroke induces the occurrence of highly heterogenous microglia

To explore the transcriptional changes in brain following ischemic injury at single cell level, we sorted cells from brain tissues around infarction of 3 day post-tMCAO or sham mice (Additional file 2: Fig. S1A), generating a thorough map of the 16 most distinctive transcription subpopulations (Additional file 2: Fig. S1B–F, Additional file 1: Table S3). Meanwhile, we found that microglia from tMCAO mice brain exhibited increased contacts with other cell types, including pericytes, endothelial cells, macrophages, oligodendrocytes, and astrocytes compared to those microglia from the sham brain (Additional file 2: Fig. S2B). We further investigated specific ligand–receptor interactions between microglia and other cell types and identified several interactions that were notably more prevalent in tMCAO brains, including Tgab-Egfr, Sema4d-Plxnb2, Ptprac-Cd2, H2m3-Cd8, Grn-Sort1, and Cd86-Cd28 (Additional file 2: Fig. S2C). Specifically, we observed increased cellular interactions between microglia and other cell types in the tMCAO mice compared to those in the sham mice, with enhanced ligand–receptor axes of Fn1-(Itga3/4^+^Itgb1), Lamc1-(Itga1^+^Itgb1), and Tgfb1-(Tgfbr1^+^Tgfbr2) among pericytes and endothelial cells (Additional file 2: Fig. S2C), as well as Fn1-(Itgav^+^Itgb1), Hspg2-Dag1, Jam2-(Itgav^+^Itgb1), Lamc1-(Itgav^+^Itgb8), Pros1-Axl, Spp1-(Itgav^+^Itgb1), and Psap-Gpr37l1 among oligodendrocyte precursor cells (OPCs) (Additional file 2: Fig. S2C). These results suggest that the enhanced cellular contacts of microglia with other cells, such as OPCs, pericytes, and endothelial cells, may play an important role in the development and progression of ischemic stroke.

**Fig. 1:**
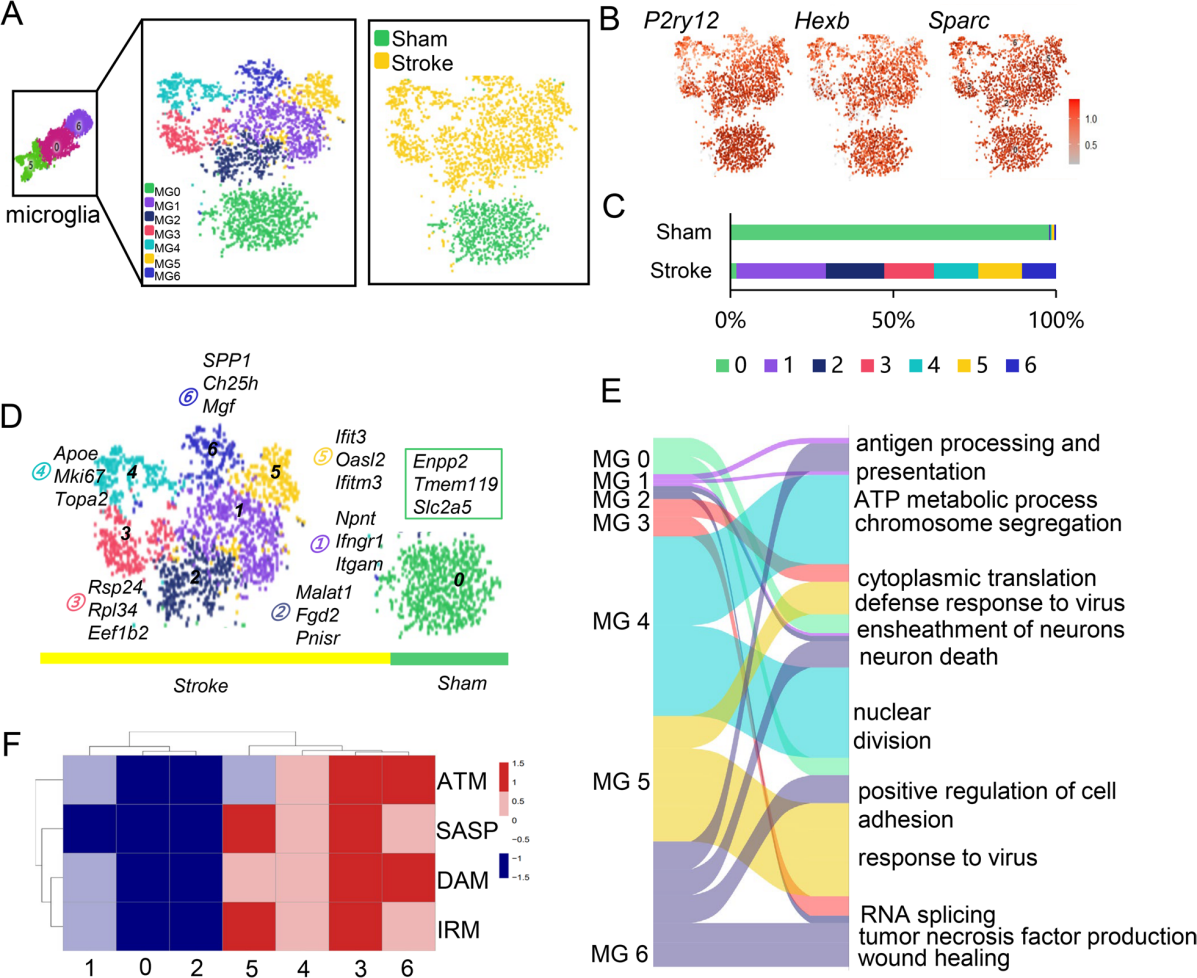
scRNA-seq revealed heterogeneity of microglial transcriptional states in the ischemic mice brain. **A** Microglia t-SNE representation. Each dot represents a single cell. **B** Expression of canonical marker genes of microglia (*P2ry12*, *Hexb*, *Sparc*). **C** Percentage of each MG cluster identified in **B** in different groups (Sham and Stroke). **D** Top highly expressed genes in MG 0–6 clusters. **E** Alluvial plot depicting the most affected GO for each cluster. Upregulated genes of each cluster were used for the enrichment analysis. Ribbon thickness indicates the number of genes per biological term. **F** Heatmap showing the GSVA score of ATM, SASP, DAM, and IRM for each cluster defined as the average normalize expression of the pathway-related genes. See Additional file 1: Table S5 for a list of DAM, IRM, IRM and ATM associated genes. (*t-SNE* t-distributed stochastic neighbor embedding, *MG* microglia, *GO* gene ontology, *GSVA* gene set variation analysis, *ATM* axon tract associated microglia, *SASP* senescence-associated secretory phenotype, *DAM* disease-associated microglia, *IRM* injury-responsive microglia)

To explore the detailed transcriptional changes of the highly heterogenous microglia after ischemic stroke, we reclassified microglia (Additional file 2: Fig. S2A) and found seven distinct microglia subclusters, MG0–MG6 (Fig. 1A), which all expressed canonical microglia genes, including *P2ry12*, *Hexb*, *Sparc,* and *Olfml3* (Fig. 1B). We defined the above MG0 to MG6 subclusters using *Slc1a2*, *Npnt*, *Malat1*, *Rpl24*, *Mki67*, *Oasl2*, and *Ch25h*, respectively (Fig. 1D). Ischemic stroke induced dramatic alteration in the proportion of microglia subclusters. In the sham brain, 98% of the microglia were MG0. While in tMCAO mice, only 2% of the microglia were MG0, 28% were MG1, 18% were MG2, 15% were MG3, 14% were MG4, 13% were MG5 and 10% were MG6 (Fig. 1C). These distinct gene expression patterns suggested that each microglia cluster exhibited previously undefined transcriptional profile [30].

To investigate the functional profiles of each cluster, we performed GO terms and KEGG pathway analysis on microglia and found that most microglia subclusters after stroke exhibited enhanced antigen processing and presentation, cell migration, and chemotaxis (Fig. 1E, Additional file 2: Fig. S3A). In MG6, we identified up-regulated genes involved in the synthesis of tumor necrosis factor, wound healing, and phagocytosis. MG3 was enriched in ribosomal components and MG4 was enriched in cell division. We also found that genes enriched in MG5 were associated with response to virus (Fig. 1E). Furthermore, we compared the transcriptional characteristics of these clusters with several previously defined microglia populations under different pathological and physiological conditions, including injury-responsive microglia (IRM) detected in lysolecithin (LPC)-induced demyelination, disease-associated microglia (DAM) found in Alzheimer's disease, axon tract associated microglia (ATM) in the developing brain, and senescence-associated secretory phenotype (SASP) identified in senescent microglia [8, 31] (Fig. 1F). We found that MG5 was close to IRM and SASP, whereas MG6 resembled ATM and DAM. Intriguingly, MG3 was remarkably comparable to the above four microglia phenotypes, which may be a result of its highly active ribosomes (Additional file 2: Fig. S3B, C). The above findings suggest that the microglia in the ischemic brain can be classified into six subclusters (MG 1–6) with distinct functional profiles.

### Identification of three microglia subclusters associated with ischemic stroke (ISAM)

**Fig. 2:**
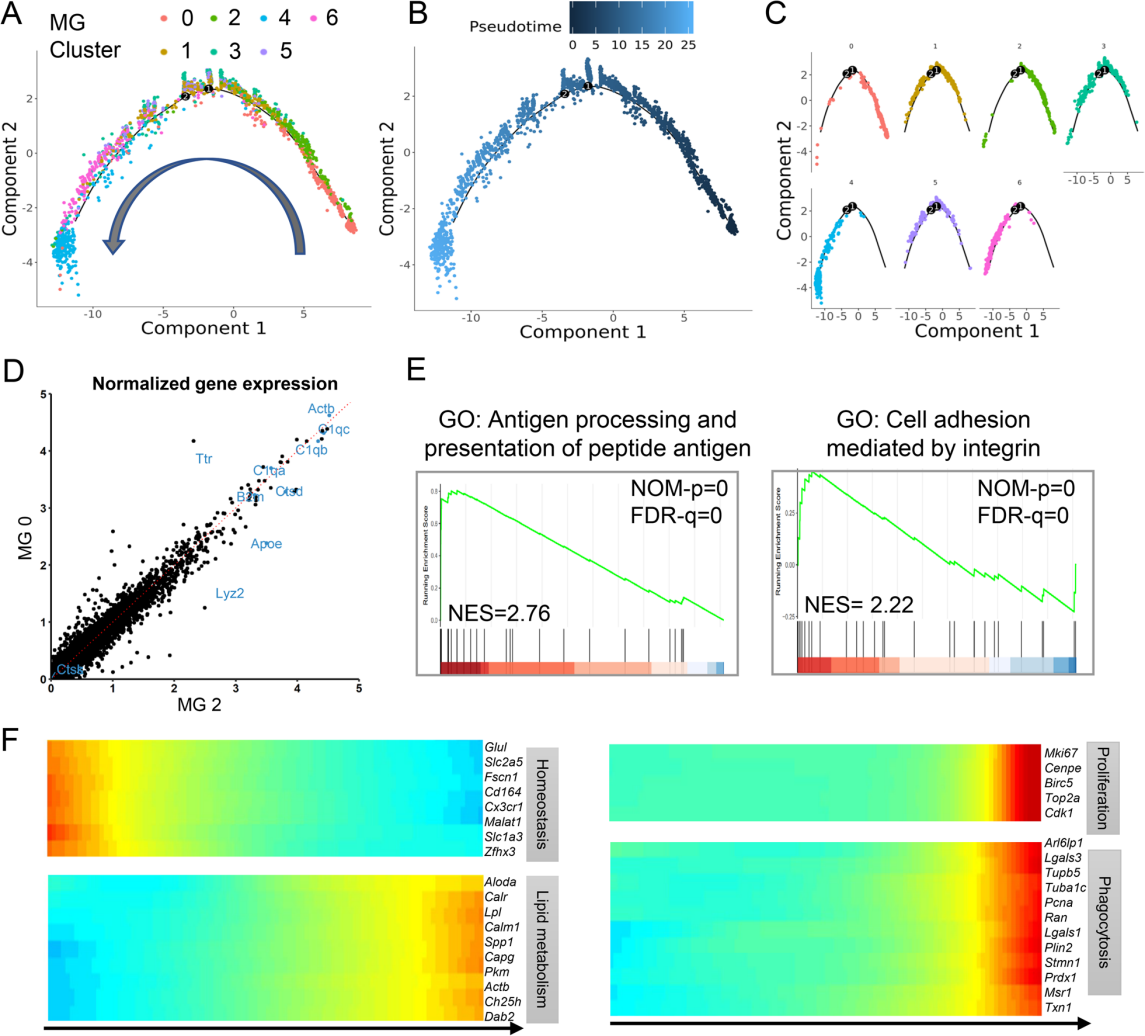
Pseudo-time trajectory revealed homeostatic microglia subcluster differentiated into ISAM. **A**–**C** Monocle pseudo-time trajectory shows the progression of MG0-MG6. One branch was identified, leading to MG4 from MG0&2 (**A**). The color represents microglia clusters (**A**, **C**), pseudo-time (**B**). **D** Comparison of normalized gene expression in MG2 and MG0. **E** GSEA using DEGs of MG2 reveals positive enrichment for antigen processing and presentation of peptide antigen and cell adhesion mediated by integrin. **F** Gene expression heatmap across pseudotime. The DEGs are shown using heatmap with microglia in pseudo-time from root to termini. Terms associated with DEGs in the right columns. (*ISAM* ischemic sociated microglia, *MG* microglia, *GSEA* gene set enrichment analysis, *DEG* differentially expressed genes)

We explored the differentiation trajectory of microglia subclusters after stroke, and found MG0 and MG2 situated at the beginning of the pseudo-time trajectory, while MG4, 5 and 6 at the end (Fig. 2A–C). MG0 was comprised of homeostatic microglia mainly from the sham mice brain, MG2 from the tMCAO group also pronouncedly expressed homeostasis-related genes (*Sall1*, *P2ry12*, *Trem119*, etc.) [10] (Additional file 2: Fig. S3E). Transcriptome comparison of MG2 and MG0 also revealed their high homogeneity (Fig. 2D). However, MG2 showed higher expression of major histocompatibility complex II (MHCII)-related genes (Additional file 2: Fig. S3F) and was enriched in antigen processing and cell adhesion pathways (Fig. 2E).

In contrast, gene expression heatmap across pseudotime revealed steady downregulation of microglial homeostasis genes (including *Cx3cr1* and *Trem119*), while lipid metabolism, proliferation, and phagocytosis-related genes (including *Trem2*, *Mki67*, and *Apoe*) were up-regulated (Fig. 2F). MG4, MG5, and MG6, which were predominantly found in the stroke brain, exhibited high expression levels of stroke-associated genes and have previously been associated with enhanced phagocytosis, lysosomal functions, and lipid metabolism [32, 33]. Therefore, we defined these three clusters (MG4, MG5 and MG6) as ischemic stroke-associated microglia.

### Proliferative MKI67^+^ microglia can differentiate into two OASL + and CH25H + subclusters

**Fig. 3:**
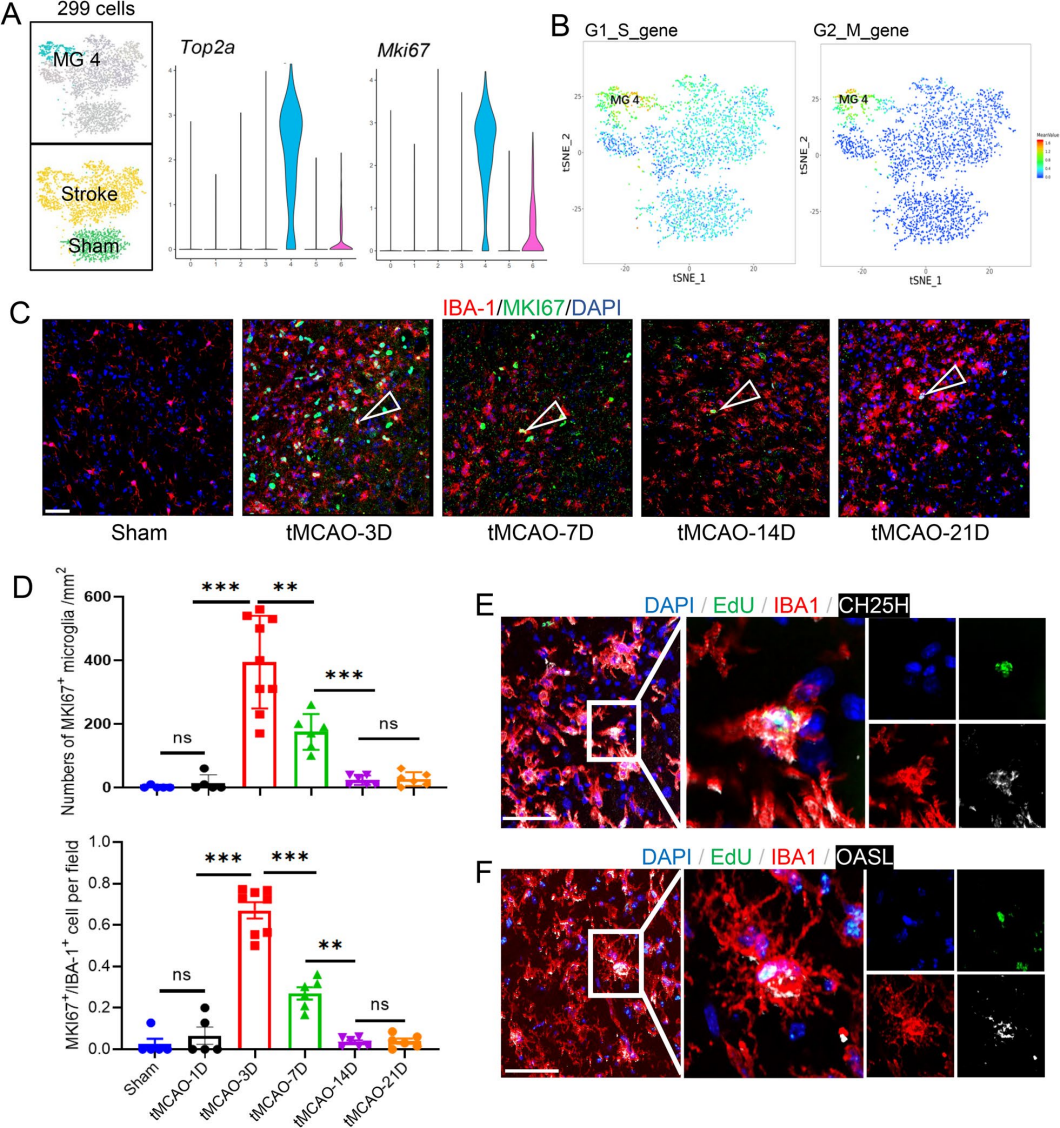
Proliferative microglia cluster emerged in the ischemic mice brain. **A** t-SNE representation of MG4 (left); violin plots showing the top up-regulated genes in MG4 (right). **B** Feature plots displaying cell cycle score determined by expression of highly conserved cell cycle genes. **C**, **D** Representative confocal images of MKI67 and IBA-1 double immunostaining in the ischemic penumbra and quantification of the MKI67^+^/IBA‐1^+^ cells at different timepoints after tMCAO. Scale bar: 40 μm (overviews). Triangles indicate resident MKI67^+^ microglia. one-way ANOVA with Bonferroni multiple comparisons test. *n* = 6–8. The data are shown as means ± SD. *p < 0.05, ***p < 0.001, n.s. no significance. **E**, **F** Representative confocal images of CH25H/OASL, IBA-1 and EdU treble immunostaining in the ischemic penumbra 3d after tMCAO. (*tMCAO* transient middle cerebral artery occlusion, *t-SNE* t-distributed stochastic neighbor embedding, *MG* microglia, *IBA-1* ionized calcium binding adapter molecule 1, *ANOVA* analysis of variance.)

MG4 highly expressed a range of cell cycle-related genes, including *MKi67* and *Top2a*, thus was defined as proliferative MKI67^+^ microglia (MG4) (Fig. 3A, B). The presence and location of this subcluster was verified using immunofluorescence labeling of MKI67, which revealed that MKI67^+^ subcluster resided in ischemic penumbra and the lateral ventricle, particularly in the subventricular zone (SVZ) 3 days after stroke (Additional file 2: Fig. S4A). Whereas at 7 days after tMCAO, the majority of MKI67^+^ microglia surrounding the ventricle disappeared. The number of MKI67^+^ microglia around the infarct zone decreased steadily and disappeared up to 21 days after tMCAO (Fig. 3C, D). These results revealed the temporal and spatial characteristic of proliferative MKI67^+^ microglia in the ischemic mice brain.

We also found that MKI67^+^ microglia shared signature genes with MG5 (OASL^+^) including *Ifitm3*, *Rtp4*, and *Ifi27l2a*, and shared signatures with MG6 (CH25H^+^), such as *Spp1*, *Lgals3*, and *Igf1* (Additional file 2: Fig. S3D), suggesting partial overlap of transcriptional features between MG4 and MG5/MG6. We regressed out cell cycle scores and compared MG4 with other clusters. After decyclicization, the distinction between MG4 and MG5, 6 disappeared (Additional file 2: Fig. S4C, D), suggesting MG4 could represent the proliferative status of MG5 and MG6. In addition, using systemic in vivo DNA labeling with 5-ethynyl-2′-deoxyuridine (EdU), we found EdU were incorporated in OASL^+^ MG5 and CH25H^+^ MG6 subclusters in the ischemic stroke brain 3 days after stroke (Fig. 3E, F, Additional file 2: Fig. S4B). These results suggest that in response to cerebral ischemia, the proliferative MKI67^+^ microglia could differentiate into CH25H^+^ and OASL^+^ subclusters.

### CH25H^+^ microglia exhibit increased phagocytosis activity after stroke

**Fig. 4:**
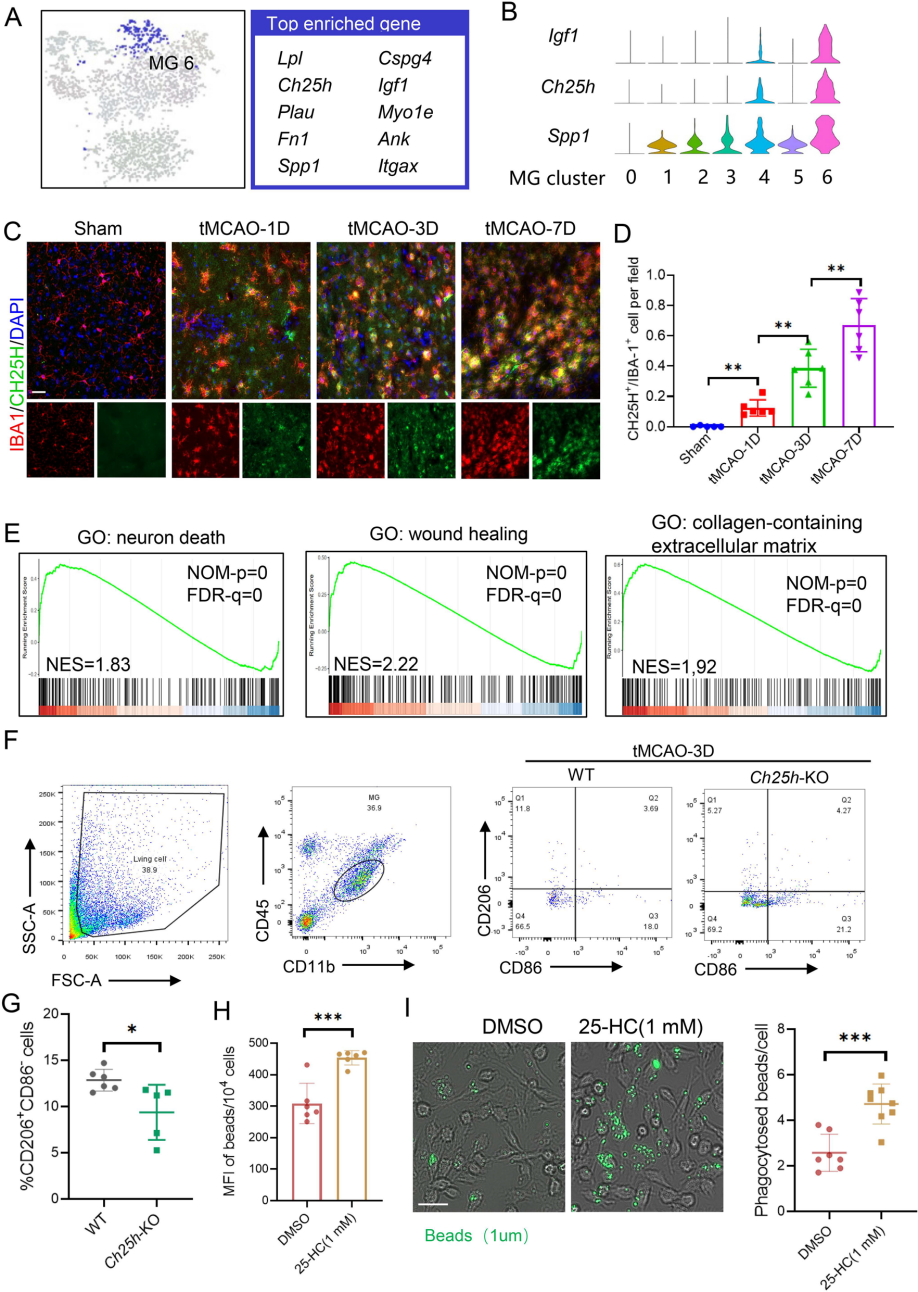
Novel CH25H^+^ microglia cluster identified with neuroprotective effect after stroke. **A** t-SNE representation and top up-regulated genes of MG6. **B** Violin plots showing specific marker of the MG6. **C**, **D** Dual immunofluorescence confirms the co-expression of CH25H with IBA1 in the ischemic penumbra and quantification of the percentage of CH25H^+^ IBA1^+^ cells at different timepoints after tMCAO (*n* = 5–6,). Scale bars, 40 μm. (*n* = 5/6 per group, one-way ANOVA with Bonferroni multiple comparisons test). The data are shown as means ± SD. *p < 0.05, ***p < 0.001, n.s. no significance. **E** GSEA using DEGs of MG6 reveals positive enrichment of neuron death, wound healing and collagen-containing extracellular matrix. **F** Flow cytometric analysis of CD206^+^M2-microglia and CD86^+^M1-microglia among CD11b^high^ CD45^int^ microglia from WT and Ch25h^−/−^mice at 3 days following tMCAO. **G** Quantification of flow cytometry of CD206^+^CD86^−^ cells in the brain at 3d after tMCAO. (**H–I**) Quantification of flow cytometry **H** and immunofluorescence of **I** internalized fluorescent latex beads. BV2 cells were subjected to hypoxia and glucose deprivation for 3 h, followed by treatment with 25-HC (1 mM) or vehicle for 6 h. And then, BV2 cells were incubated with the beads at a ratio of 1:10 for 2 h, followed by treatment washed and fixed for analysis. Scale bar: 50 μm. (*t-SNE* t-distributed stochastic neighbor embedding, *MG* microglia, *CH25H* Cholesterol 25-Hydroxylase, *IBA-1* ionized calcium binding adapter molecule 1, *ANOVA* analysis of variance, *QuSAGE* quantitative set analysis for gene expression, *GSEA* gene set enrichment analysis, *DEG* differentially expressed genes, *25-HC* 25-hydroxycholesterol.)

By examining the molecular features of CH25H^+^ MG6, we found several genes in MG6 were enriched in proliferative-region-associated microglia [34] or DAM [10], such as *Spp1*, *Gpnmb*, *Igf1* and *Ctsb*. In addition, CH25H^+^ MG6 also exhibited up-regulated extracellular matrix (ECM)-related genes (for example, *Fn1* and *Thbs1*) and fatty acid-related genes, including *Lpl* and *Ch25h* (Fig. 4A, B). The cholesterol 25-hydroxylase (*Ch25h*) was specifically expressed in MG6 (Additional file 1: Table S6), and CH25H^+^ IBA1^+^ cells could be found in the ischemic penumbra starting from 1 day and gradually increased up to 7 days after stroke (Fig. 4C, D). Moreover, MG6 showed gene enrichment in neuron death, phagocytosis, wound healing and collagen containing extracellular matrix (Fig. 4E). We further found that CH25H^+^ MG6 showed the highest anti-inflammatory score, with high expression of phagosome associated genes and de novo synthesis of fatty acid genes (Additional file 2: Fig. S5A). Our flow cytometry analysis revealed a significant decrease of CD206^+^ microglia in the brains of Ch25h^−/−^ mice, suggesting that *Ch25h* may play a role in regulating microglial phenotype (Fig. 4F, G). Moreover, we conducted flow cytometry (Additional file 2: Fig. S5B) and immunofluorescence experiments (Fig. 4I) in vitro, in which we treated microglial cell line BV2 cells with 25-hydroxycholesterol (25-HC), a metabolite of CH25H, and found increased phagocytic ability (Fig. 4H, I. These findings suggest that *Ch25h*, which is specifically expressed in CH25H^+^ MG6, plays a critical role in the phagocytic ability of microglia after ischemic stroke and accounts for the neuroprotective and anti-inflammatory properties of microglia after stroke.

### *Ch25h* deficiency exacerbates ischemic brain injury and neuroinflammation after stroke

**Fig. 5:**
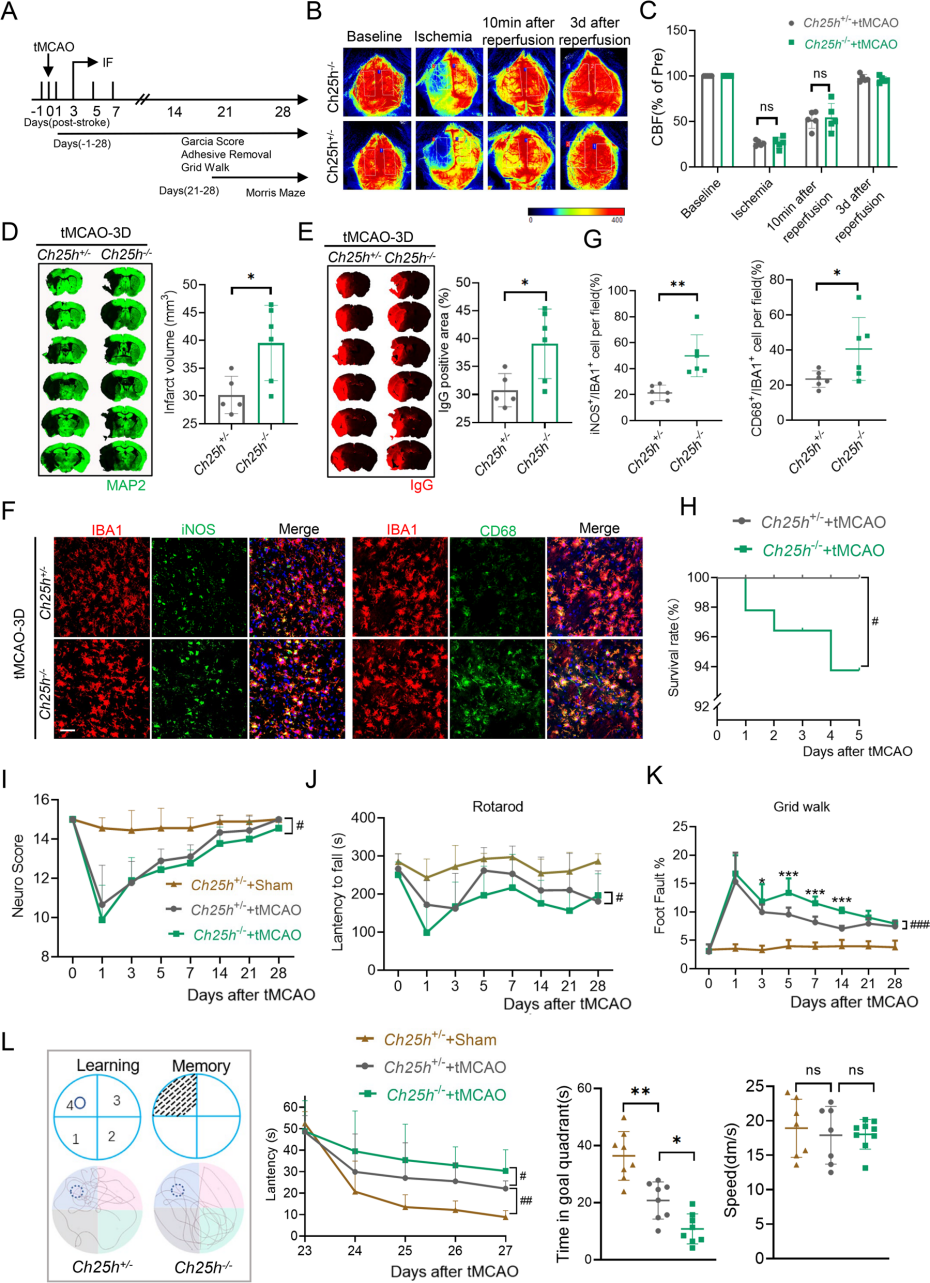
*Ch25h* deficiency exacerbates ischemic brain injury and neuroinflammation after stroke. **A** Schematic representation of the experimental design. **B** Cerebral blood flow monitored using two-dimensional laser speckle imaging techniques before, during middle cerebral artery occlusion (tMCAO), 10 min and 3 d after reperfusion. **C** Results of **B** were expressed as percent change from baseline (pre-tMCAO). **D** Representative MAP2 staining of brain infarct and quantification of infarct volume 3 days after stroke in *Ch25h*^+/−^and *Ch25h*^−/−^mice. *n* = 5–6 per group. **E** Representative endogenous mouse IgG staining of BBB leakage and quantification of endogenous IgG positive area 3 days after stroke in *Ch25h*^±^and *Ch25h*^−/−^mice. *n* = 5–6 per group. **F** Immunostaining of iNOS/CD68 and IBA1 in brain Section 3 days after tMCAO from *Ch25h*^+/−^and *Ch25h*^−/−^mice. Scale bar = 40 μm. **G** Quantification of the percentage of iNOS^+^/IBA1^+^ and CD68^+^/IBA1^+^ cells in the ischemic brain, *n* = 5–6 per group. **H** Survival rate of *Ch25h*^+/−^and *Ch25h*^−/−^mice within 5 days after tMCAO. ^#^*P* ≤ 0.05(*Ch25h*^+/−^ + tMCAO vs *Ch25h*^−/−^ + tMCAO). **I** Sensorimotor functions were assessed using the Garcia Score. **J** Sensorimotor functions were assessed using the rotarod test. **K** Sensorimotor functions were assessed using the Grid walk. *n* = 8–10. **L** Cognitive functions were evaluated in the Morris water maze. Representative images show the swim paths. *n* = 8–10. *P ≤ 0.05, **P ≤ 0.01, ***P ≤ 0.001 (*Ch25h*^+/−^ + tMCAO vs *Ch25h*^−/−^ + tMCAO at 3d, 5d, 7d, 14d, 28d), ^#^*P* ≤ 0.05, ^##^*P* ≤ 0.01, ^####^*P* ≤ 0.0001 (*Ch25h*^+/−^ + tMCAO vs *Ch25h*^−/−^ + tMCAO), two‐way ANOVA, and post hoc Tukey's tests. (*MG* microglia, *CH25H* Cholesterol 25-Hydroxylase, *IBA-1* ionized calcium binding adapter molecule 1, *iNOS* inducible nitric oxide synthase)

To decipher the role of *Ch25h* in microglia after ischemic stroke, we subjected *Ch25h*^−/−^ mice to 60-min tMCAO, giving the finding that *Ch25h* was mainly expressed in microglia of ischemic stroke brain (Additional file 2: Fig. S2D, E). We found the extent of cerebral blood flow reduction was equivalent between the two groups (Fig. 5A–C). We found that the *Ch25h* gene of the heterozygous was consistent with that of wild type mice as revealed by PCR and immunostaining; therefore, *Ch25h*^+/−^ mice was used as the control group (Additional file 2: Fig. S5C, D). The total infarct volume at 72 h following tMCAO were significantly increased in the *Ch25h*^−/−^ mice compared to the *Ch25h*^+/−^ mice (Fig. 5D). The blood brain barrier (BBB) disruption was exacerbated in *Ch25h*^−/−^ mice compared to *Ch25h*^+/−^ mice after stroke (Fig. 5E). Consistent with the lesion size, the neuroinflammatory iNOS^+^ IBA1^+^ and CD68^+^ IBA1^+^ microglia in the ischemic penumbra were significantly increased in *Ch25h*^−/−^ mice (Fig. 5F, G). In addition, we found reduced survival of *Ch25h*^−/−^ mice compared to the *Ch25h*^+/−^ mice within 5 days after tMCAO (Fig. 5H). We next investigated the sensorimotor functions using modified Garcia score (Fig. 5I), the rotarod test (Fig. 5J) and grid‐walking test (Fig. 5K). We found prolonged latency to fall and increased foot fault percentage in *Ch25h*^−/−^ mice compared to *Ch25h*^+/−^ mice 5 days after stroke. In the Morris water maze test, which assesses the spatial cognitive functions, we found *Ch25h*^−/−^ mice manifested an increased latency to find the hidden platform and reduced time spent in the goal quadrant after the platform was removed (Fig. 5L), indicating impaired spatial learning and memory retention, respectively. There was no difference in swimming speed between *Ch25h*^−/−^ and *Ch25h*^+/−^ mice. Collectively, these results support that *Ch25h* deficiency exacerbates ischemic brain injury, neuroinflammation and functional recovery after tMCAO.

### OASL^+^ microglia subcluster exhibits upregulated type I interferon signaling in the post-stroke mice brain

**Fig. 6:**
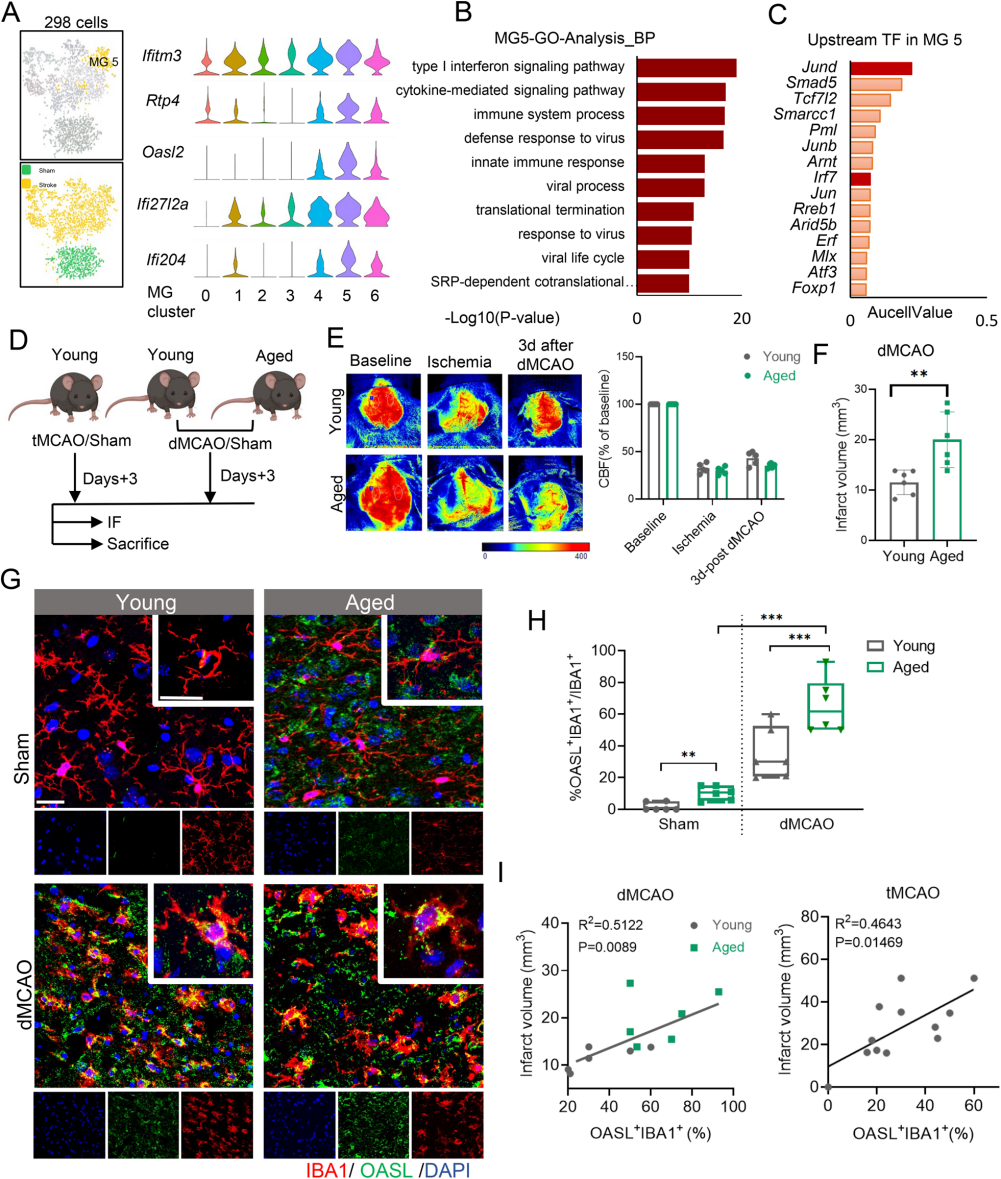
OASL^+^ microglia subset exhibits type I interferon upregulated signaling and was associated with increased infarct volume after stroke. **A** t-SNE representation of MG5 (left); violin plots showing the top up-regulated genes in MG5 (right). **B** Enriched GO terms in MG5. **C** Top predicted upstream transcriptional regulators of MG5 activation. **D** Schematic representation of the experimental design. **E** Cerebral blood flow was monitored using two-dimensional laser speckle imaging techniques on the left side before, during, and 3 days after distal middle cerebral artery occlusion (dMCAO). The values on the right side were expressed as a percentage change from the baseline measurements taken prior to dMCAO. **F** Quantification of infarct volume 3 days after dMCAO of young (3 months) and aged (21 months) mice. *n* = 6 per group. **G** Representative images of microglia in adult brain and aged brain after ischemic stroke. Scale bars, 30 μm. **H** OASL^+^ quantified in microglia (IBA1^+^) in the brain of young (3 months) and aged (21 months) mice of sham and ischemic stroke mice (young, *n* = 12; aged, *n* = 12; two-sided *t* test; mean ± SD). **I** Correlation between OASL^+^ microglia proportion and infarct volume of tMCAO and dMCAO. Data are presented as means ± SD. (*t-SNE* t-distributed stochastic neighbor embedding, *MG* microglia, *GO* gene ontology, *DEG* differentially expressed genes, *IBA-1* ionized calcium binding adapter molecule 1, *OASL* 2'–5'-oligoadenylate synthetase-like, *tMCAO* transient middle cerebral artery occlusion, *dMCAO* distal middle cerebral artery occlusion; *ANOVA* analysis of variance)

We next found OASL^+^ MG5 showed unique expression of interferon response genes including interferon induced transmembrane protein 3 (*Ifitm3*), receptor transporter protein 4 (*Rtp4*), 2′5′ oligoadenylate synthetase-like 2 (*Oasl2*), alpha-inducible protein 27-like 2A (*Ifi27l2a*), and *Ifi204* (Fig. 6A). Functional analysis of MG5 also demonstrated gene enrichment in type I interferon (IFN)-related pathways and innate immune response (Fig. 6B, Additional file 2: Fig. S6A).

Using SCENIC analysis, we found that *Irf7* and *Jund* were upstream transcription factors responsible for transcriptomic alterations in OASL^+^ MG5, with the expression of *Irf7* specifically elevated in MG5 (Fig. 6C, Additional file 2: Fig. S6B). We further predicted a large repertoire of DEGs regulated by *Irf7*, suggesting that *Irf7* was a main regulator of ischemic-mediated activation of interferon response in microglia (Additional file 2: Fig. S6C). We further found that OASL^+^ IBA1^+^ cells were localized in the peri-infarct area in the cortex and striatum of the ischemic brain (Additional file 2: Fig. S6D, F). These data suggest that cerebral ischemic stroke induced the occurrence of type I interferon signaling-related OASL^+^ microglia*.*

### OASL^+^ microglia accumulated in the aged ischemic mice brain and was associated with increased infarct volume after stroke

As mentioned above, OASL^+^ microglia exhibited an abundance of senescence-related genes (Fig. 1F). To understand the association of OASL^+^ microglia with aging, we analyzed the interferon response genes of microglia isolated from 3- to 21-month-old C57BL/6 J mice brain (GSE127893). We detected higher expression of interferon response genes in microglia from the aged mice compared to those from the young mice (Additional file 2: Fig. S7A). In addition, we found a positive association between age and interferon response gene expression in microglia from the human parietal cortices (GSE135437) (Additional file 2: Fig. S7B). By analyzing the scRNA-seq data of Li et al.[35], we identified the OASL^+^ microglia (characterized by interferon responsive genes) in the aged ischemic mice brain, the proportion of which was considerably increased compared to that in the adult ischemic brain (Additional file 2: Fig. S7C, D). After inducing dMCAO in both young and aged mice, there was no difference in blood flow between the two groups. However, after 3 day post-dMCAO, the cerebral blood flow of the aged mice exhibited a slight decrease, while the the infarct volume of the aged mice were significantly increased in the aged mice compared to that of the young mice (Fig. 6E, F). Using immunofluorescent staining, we found consistently increased proportion of OASL^+^IBA1^+^ microglia in aged mice compared to the young mice after dMCAO (Fig. 6G, H). Moreover, the proportion of OASL^+^IBA1^+^ microglia was positively correlated with the infarct volume both in the aged and young mice after tMCAO and dMCAO (Fig. 6I). These findings suggest that ischemic stroke induced the increase of the interferon-responsive OASL^+^ microglia, which was associated with the infarct volume growth and counted more in the aged brain than in young individuals.

## Discussion

Here, we identified six distinct microglia subclusters as they progressed from homeostatic state to three unique microglia subclusters in the ischemic brain. We termed the above three microglia subclusters as ISAM, including MKI67^+^, CH25H^+^, and OASL^+^ microglia. We further found that CH25H^+^ microglia could be a neuroprotective subcluster displaying increased phagocytic and healing-promoting features, while the OASL^+^ microglia was a pro-inflammatory subcluster with upregulated interferon response and senescence-related features. The current findings suggest that ISAM represent specialized and unique types of microglia that could be precisely targeted for the development of novel stroke therapeutic strategies.

The CH25H^**+**^ microglia (MG6) express several neurotrophic factors, including *Spp1*, *Gpnmb*, and *Lgals3*, which was previously suggested to induce axon outgrowth and establish functional synapses, thus may contribute to neurogenesis after stroke [36, 37]. Moreover, the CH25H^**+**^ microglia also express similar signature genes as those neural network construction associated microglia found in neonatal mice, for example, *Spp1*, *Gpnmb*, *Igf1*, *Cd68* [8, 34]*,* suggesting CH25H^**+**^ microglia could contribute to the post-stroke neural network reconstruction during brain repair.

CH25H is an enzyme that catalyzes the oxidation of cholesterol to form the soluble product 25-hydroxycholesterol (25-HC) [38], which is an oxysterol that can play an important role in different pathological process, including antiviral effects by inhibiting membrane fusion in T cells, decreasing myocardial apoptosis by inhibiting PARP activity in myocardiocytes and maintaining mitochondrial integrity and preventing spurious AIM2 inflammasome activation in macrophages [38–40]. These findings suggest the *Ch25h* in microglia may play a protective role through a plethora of mechanisms. Consistently, depletion of *Ch25h* in cerebral ischemic stroke mice exacerbated neuroinflammation, increased infarct volume and deteriorated neurological functions. Thus, *Ch25h* could be an intriguing target for modulating the protective microglia subcluster for stroke therapy.

In addition to the CH25H^**+**^ subcluster, we found that the OASL^+^ microglia was associated with upregulated interferon response. Consistently, it was previously found that ischemic brain injury induced interferon-related genes in microglia [41]. Interferon responsive genes may influence numbers of neuroinflammation-related diseases, such as MS, AD, CNS lupus and viral encephalitis [42–45]. Moreover, we found that the percentage of OASL^+^ microglia was significantly associated with cerebral infarct volume after ischemia, suggesting that OASL^+^ microglia may be a critical player of neuroinflammation after ischemic stroke.

It was previously suggested that interferon-response microglia increased during aging [46]. In our study, we found that the proportion of interferon-related OASL^+^ microglia was considerably increased in aged mice compared to adult mice after ischemic stroke. This suggests that the interferon-related microglia subcluster may contribute to age-dependent neurodegeneration and predispose individuals to various age-related brain disorders.

While our analysis provides novel insights into the transcriptional patterns of distinct microglia subclusters in the ischemic brain, it has some limitations as follows. The brain tissue collected for analysis was restricted to the ischemic penumbra and the infarct region. The transcriptional changes in other brain regions, for example, the hippocampus was less considered. The single sample collecting timepoint can only offer a snapshot of the microglia transcriptional state, leading to limited understanding of the temporal alterations of microglia subclusters after stroke [9]. In addition, the current single-cell isolation technique fails to provide spatial characteristics of the newly identified microglia subclusters. Future studies using spatial transcriptomics with single-cell resolution at different timepoints after stroke may overcome these limitations [47]. In addition, it is important to note that the findings of this study were based on the use of only male animals, and caution should be exercised when applying these findings to female animals. Biological differences between male and female animals, such as hormonal fluctuations, reproductive status, and genetic factors [4], may influence the response to stroke and the role of CH25H^+^ microglia. Therefore, further studies specifically involving female animals are warranted to better understand the potential sex differences in the contribution of CH25H^+^ microglia to stroke progression. Furthermore, while using *Ch25h*-KO mice may not be able to distinguish the role of CH25H^**+**^ microglia in stroke, more specific and targeted approaches to selectively eliminate or inactivate the CH25H^**+**^ microglia would be necessary to establish a causal relationship between the sub-cluster and stroke progression.

In conclusion, we unravel the previously undefined microglia heterogeneity following ischemic stroke and define three distinct ISAM with specific functional features in the progression of ischemic brain injury. In the future, targeting different ISAM subcluster might represent an attractive and druggable approach to decrease neuroinflammation and restore brain homeostasis, with the goal of improving neurological functions after ischemic stroke.

## Supplementary Information

**Additional** **file** **1. **Additional tables.

**Additional** **file** **2. **Additional figures and methods.

## Data Availability

All data sets are included in the manuscript and supporting information. Original data are available upon request.
