# Supplementary material for: Novel CH25H+ and OASL+ microglia subclusters play distinct roles in cerebral ischemic stroke
Source: J Neuroinflammation. 2023 May 15;20:115. doi: 10.1186/s12974-023-02799-6 (PMC10184422; doi:10.1186/s12974-023-02799-6)
Supplement: Supplementary file 1 — Additional file 1. Additional tables. [file 12974_2023_2799_MOESM1_ESM.docx]

**Additional file 1**

**Novel CH25H^+^ and OASL^+^ Microglia Subclusters Play Distinct Roles in Cerebral Ischemic Stroke**

Yueman Zhang^#1^, Yunlu Guo^#1^, Ruqi Li^2^, Tingting Huang^1^, Yan Li^1^, Wanqin Xie^1^, Chen Chen^1^, Weijie Chen^1^, Jieqing Wan^2^, Weifeng Yu^1^, Peiying Li*^1,3^

^1^ Department of Anesthesiology, Key Laboratory of the Ministry of Education, Renji Hospital, Shanghai Jiao Tong University School of Medicine, Shanghai, China

^2^Department of Neurological Surgery, Renji Hospital, Shanghai Jiao Tong University School of Medicine, Shanghai, China

^3^Clinical Research Center, Renji Hospital, Shanghai Jiao Tong University School of Medicine, Shanghai, China

Running Title: Ischemic stroke associated microglia

*Correspondence:

Dr. Peiying Li, MD, PhD.

Department of Anesthesiology, Clinical Research Center

Renji Hospital, Shanghai Jiao Tong University School of Medicine

Shanghai, China; 160 Pujian Rd

Shanghai 200127, China

Email: peiyingli.md@gmail.com

Phone: 8615800616866

#These authors contributed equally to this work.

**Tables**

| **Table S2：List of antibodies** | | |
| --- | --- | --- |
| **Antibodies** | **Source** | **Identifier** |
| goat anti-Iba-1 | Abcam | ab178846 |
| rabbit anti- MAP2 | santa cruz | L1714 |
| rat anti‐Mki67 | eBioscience™ | 14-5698-821 |
| Rabbit anti‐OASL | abcam | ab191701 |
| Rabbit anti‐iNOS | abcam | ab191701 |
| Mouse anti-CH25H | Novus Biologicals | H00009023 |
| Rat-anti-CD68 | abcam | ab53444 |
| anti-CD11b-FITC | Biolegend | 101206 |
| anti-CD86-BV510 | Biolegend | 105040 |
| anti-CD45-APC-Cy7 | Biolegend | 103116 |
| anti-CD206-APC | Biolegend | 141708 |
| Donkey Anti-Rabbit IgG H&L (Alexa Fluor® 594) | abcam | ab150076 |
| Donkey Anti-Rat IgG H&L (Alexa Fluor® 594) | abcam | ab150156 |
| Donkey Anti-Goat IgG H&L (Alexa Fluor® 594) | abcam | ab150136 |
| Donkey Anti-Mouse IgG H&L (Alexa Fluor® 488) | abcam | ab150105 |
| Donkey Anti-Rabbit IgG H&L (Alexa Fluor® 488) | abcam | ab150073 |
| Donkey Anti-Rat IgG H&L (Alexa Fluor® 488) | abcam | ab150153 |

| **Table S3：Celltypes and Mainmarkers of brain cells** | | |
| --- | --- | --- |
| Cluster | Cell type | Main markers |
| 0 | Microglia | Ptprc, P2ry12, Sall1 |
| 1 | Astrocyte | Aldoc, Aqp4 |
| 2 | Smooth muscle cell | Tagln, Acta2 |
| 3 | Macrophage | Ptprc, Ms4a7 |
| 4 | Smooth muscle cell | Tagln, Acta2 |
| 5 | Microglia | Ptprc, P2ry12, Sall1 |
| 6 | Microglia | Ptprc, P2ry12, Sall1 |
| 7 | Pericyte | Kcnj8 |
| 8 | Endothelial | Cldn5, Flt1, Pecam1 |
| 9 | Astrocyte | Aldoc, Aqp4 |
| 10 | Oligodendrocyte | Mbp, Sox10 |
| 11 | Neutrophil | S100a9, Csf3r |
| 12 | Excitatory neuron | Snap25, Slc17a7, ALDOC |
| 13 | OPC | Pdgfra |
| 14 | Oligodendrocyte | Mbp, Sox10 |
| 15 | Undefined |  |
| 16 | Ependymal | Foxj1, Ccdc153, Enkur |

**Table S4：Differentially expressed genes in** microglia subclusters

| allmarkers.gene | p_val | avg_logFC | pct.1 | pct.2 | DifPct | p_val_adj | cluster |
| --- | --- | --- | --- | --- | --- | --- | --- |
| Aldoc | 1.29E-92 | 0.765064 | 0.571 | 0.262 | 0.309 | 3.32E-88 | 0 |
| Mt3 | 1.06E-75 | 0.635595 | 0.413 | 0.14 | 0.273 | 2.71E-71 | 0 |
| Ntsr2 | 4.40E-62 | 0.617336 | 0.431 | 0.182 | 0.249 | 1.13E-57 | 0 |
| Mobp | 4.65E-62 | 1.07465 | 0.496 | 0.245 | 0.251 | 1.19E-57 | 0 |
| Ttyh1 | 1.32E-61 | 0.579664 | 0.455 | 0.201 | 0.254 | 3.38E-57 | 0 |
| Gnao1 | 8.60E-60 | 0.57914 | 0.413 | 0.17 | 0.243 | 2.21E-55 | 0 |
| Fam107a | 1.93E-59 | 0.554262 | 0.357 | 0.124 | 0.233 | 4.96E-55 | 0 |
| Plpp3 | 1.44E-58 | 0.648905 | 0.463 | 0.223 | 0.24 | 3.69E-54 | 0 |
| Gjb6 | 2.21E-55 | 0.553307 | 0.34 | 0.12 | 0.22 | 5.68E-51 | 0 |
| Ndrg2 | 2.22E-52 | 0.585259 | 0.42 | 0.198 | 0.222 | 5.71E-48 | 0 |
| Htra1 | 1.91E-49 | 0.532462 | 0.335 | 0.129 | 0.206 | 4.90E-45 | 0 |
| Gja1 | 5.12E-49 | 0.587526 | 0.391 | 0.182 | 0.209 | 1.31E-44 | 0 |
| Nfasc | 4.57E-48 | 0.526045 | 0.438 | 0.226 | 0.212 | 1.17E-43 | 0 |
| Top2a | 1.35E-283 | 2.219657 | 0.86 | 0.092 | 0.768 | 3.47E-279 | 4 |
| Mki67 | 1.92E-283 | 2.242191 | 0.9 | 0.111 | 0.789 | 4.93E-279 | 4 |
| Hmmr | 1.08E-275 | 1.308046 | 0.659 | 0.033 | 0.626 | 2.77E-271 | 4 |
| Nusap1 | 6.17E-272 | 1.086936 | 0.599 | 0.022 | 0.577 | 1.58E-267 | 4 |
| Kif23 | 1.09E-269 | 0.942128 | 0.565 | 0.018 | 0.547 | 2.81E-265 | 4 |
| Cenpe | 5.79E-269 | 1.437606 | 0.686 | 0.041 | 0.645 | 1.49E-264 | 4 |
| Bub1 | 4.12E-267 | 0.807147 | 0.582 | 0.021 | 0.561 | 1.06E-262 | 4 |
| Cdk1 | 2.08E-266 | 1.432923 | 0.803 | 0.078 | 0.725 | 5.35E-262 | 4 |
| Kif11 | 1.79E-261 | 0.781424 | 0.528 | 0.014 | 0.514 | 4.59E-257 | 4 |
| Tpx2 | 4.67E-257 | 1.406532 | 0.726 | 0.058 | 0.668 | 1.20E-252 | 4 |
| Ccna2 | 4.77E-256 | 1.245329 | 0.709 | 0.052 | 0.657 | 1.23E-251 | 4 |
| Knl1 | 1.31E-252 | 0.769658 | 0.495 | 0.011 | 0.484 | 3.37E-248 | 4 |
| Pclaf | 7.30E-252 | 1.343113 | 0.732 | 0.061 | 0.671 | 1.87E-247 | 4 |
| Kif15 | 1.70E-250 | 0.942912 | 0.609 | 0.03 | 0.579 | 4.36E-246 | 4 |
| Racgap1 | 3.49E-242 | 1.024556 | 0.662 | 0.046 | 0.616 | 8.96E-238 | 4 |
| Nuf2 | 8.53E-241 | 0.946093 | 0.612 | 0.035 | 0.577 | 2.19E-236 | 4 |
| Ckap2l | 6.11E-239 | 0.968757 | 0.579 | 0.028 | 0.551 | 1.57E-234 | 4 |
| Smc4 | 4.90E-237 | 1.610388 | 0.87 | 0.125 | 0.745 | 1.26E-232 | 4 |
| Ndc80 | 3.35E-234 | 0.657432 | 0.482 | 0.013 | 0.469 | 8.61E-230 | 4 |
| Cdca8 | 7.64E-234 | 1.083361 | 0.645 | 0.045 | 0.6 | 1.96E-229 | 4 |
| Cenpf | 3.70E-227 | 1.432425 | 0.662 | 0.053 | 0.609 | 9.49E-223 | 4 |
| Pbk | 1.11E-226 | 1.016244 | 0.599 | 0.037 | 0.562 | 2.84E-222 | 4 |
| Smc2 | 1.12E-225 | 1.326671 | 0.793 | 0.093 | 0.7 | 2.87E-221 | 4 |
| Dlgap5 | 1.72E-225 | 0.721554 | 0.498 | 0.018 | 0.48 | 4.42E-221 | 4 |
| Birc5 | 2.48E-225 | 1.491909 | 0.813 | 0.108 | 0.705 | 6.36E-221 | 4 |
| Plk1 | 5.76E-224 | 0.933631 | 0.552 | 0.028 | 0.524 | 1.48E-219 | 4 |
| Kif20b | 9.53E-223 | 0.912954 | 0.562 | 0.03 | 0.532 | 2.45E-218 | 4 |
| Ckap2 | 2.81E-219 | 0.875119 | 0.542 | 0.027 | 0.515 | 7.21E-215 | 4 |
| Hmgb2 | 2.14E-217 | 1.057763 | 0.712 | 0.069 | 0.643 | 5.49E-213 | 4 |
| Prr11 | 2.28E-217 | 0.928008 | 0.545 | 0.029 | 0.516 | 5.86E-213 | 4 |
| Prc1 | 1.72E-215 | 0.837663 | 0.462 | 0.014 | 0.448 | 4.41E-211 | 4 |
| Mis18bp1 | 3.54E-206 | 0.74621 | 0.468 | 0.018 | 0.45 | 9.09E-202 | 4 |
| Aspm | 6.32E-204 | 0.824374 | 0.462 | 0.018 | 0.444 | 1.62E-199 | 4 |
| Spc24 | 4.22E-203 | 0.792127 | 0.548 | 0.035 | 0.513 | 1.08E-198 | 4 |
| Ncapd2 | 1.30E-201 | 0.871443 | 0.679 | 0.068 | 0.611 | 3.33E-197 | 4 |
| Cdca3 | 1.65E-201 | 0.825387 | 0.589 | 0.044 | 0.545 | 4.24E-197 | 4 |
| Cdca2 | 7.32E-201 | 0.614463 | 0.415 | 0.011 | 0.404 | 1.88E-196 | 4 |
| Aurkb | 3.70E-199 | 0.728054 | 0.495 | 0.025 | 0.47 | 9.50E-195 | 4 |
| Cdc20 | 2.07E-198 | 0.632681 | 0.438 | 0.015 | 0.423 | 5.30E-194 | 4 |
| Gm4739 | 2.10E-198 | 0.766394 | 0.548 | 0.035 | 0.513 | 5.40E-194 | 4 |
| Ccnb1 | 4.33E-198 | 0.886824 | 0.508 | 0.028 | 0.48 | 1.11E-193 | 4 |
| Cep55 | 6.97E-192 | 0.647506 | 0.411 | 0.013 | 0.398 | 1.79E-187 | 4 |
| Cks1b | 2.71E-191 | 0.961227 | 0.719 | 0.089 | 0.63 | 6.97E-187 | 4 |
| Anln | 1.33E-189 | 0.72813 | 0.498 | 0.028 | 0.47 | 3.41E-185 | 4 |
| Tacc3 | 7.13E-189 | 0.872486 | 0.662 | 0.072 | 0.59 | 1.83E-184 | 4 |
| Bub1b | 1.50E-188 | 0.582304 | 0.458 | 0.021 | 0.437 | 3.85E-184 | 4 |
| Incenp | 1.47E-187 | 1.048478 | 0.732 | 0.098 | 0.634 | 3.77E-183 | 4 |
| Kif22 | 5.72E-187 | 0.546395 | 0.438 | 0.018 | 0.42 | 1.47E-182 | 4 |
| Rad51ap1 | 1.60E-186 | 0.614773 | 0.475 | 0.025 | 0.45 | 4.12E-182 | 4 |
| Spc25 | 1.40E-183 | 0.720471 | 0.482 | 0.028 | 0.454 | 3.59E-179 | 4 |
| H2afx | 8.18E-183 | 1.080543 | 0.696 | 0.087 | 0.609 | 2.10E-178 | 4 |
| Cit | 1.21E-182 | 0.72936 | 0.532 | 0.038 | 0.494 | 3.10E-178 | 4 |
| Trim59 | 1.27E-181 | 0.60257 | 0.465 | 0.024 | 0.441 | 3.26E-177 | 4 |
| Asf1b | 1.30E-178 | 0.557446 | 0.468 | 0.026 | 0.442 | 3.34E-174 | 4 |
| Troap | 3.61E-177 | 0.583362 | 0.398 | 0.014 | 0.384 | 9.26E-173 | 4 |
| Nek2 | 6.83E-176 | 0.537332 | 0.368 | 0.01 | 0.358 | 1.75E-171 | 4 |
| Rrm2 | 4.12E-175 | 1.169042 | 0.585 | 0.059 | 0.526 | 1.06E-170 | 4 |
| Tk1 | 4.13E-174 | 0.998598 | 0.692 | 0.092 | 0.6 | 1.06E-169 | 4 |
| Sgo1 | 2.16E-173 | 0.509614 | 0.378 | 0.012 | 0.366 | 5.54E-169 | 4 |
| Rrm1 | 2.45E-172 | 0.972264 | 0.692 | 0.092 | 0.6 | 6.30E-168 | 4 |
| Cenpa | 7.90E-172 | 1.238072 | 0.676 | 0.091 | 0.585 | 2.03E-167 | 4 |
| Ccnb2 | 1.18E-168 | 0.947941 | 0.582 | 0.059 | 0.523 | 3.04E-164 | 4 |
| Kif4 | 1.25E-164 | 0.526537 | 0.395 | 0.017 | 0.378 | 3.22E-160 | 4 |
| Kifc1 | 2.18E-164 | 0.543321 | 0.378 | 0.015 | 0.363 | 5.59E-160 | 4 |
| Ccnf | 6.43E-164 | 0.527984 | 0.361 | 0.012 | 0.349 | 1.65E-159 | 4 |
| Cdkn2c | 6.62E-164 | 0.680658 | 0.538 | 0.048 | 0.49 | 1.70E-159 | 4 |
| Uhrf1 | 2.78E-163 | 0.742126 | 0.492 | 0.038 | 0.454 | 7.12E-159 | 4 |
| Ezh2 | 4.73E-160 | 0.805952 | 0.602 | 0.069 | 0.533 | 1.21E-155 | 4 |
| Arhgef39 | 4.89E-160 | 0.58114 | 0.411 | 0.022 | 0.389 | 1.25E-155 | 4 |
| C330027C09Rik | 1.68E-157 | 0.587369 | 0.401 | 0.021 | 0.38 | 4.31E-153 | 4 |
| Cenpn | 1.73E-157 | 0.508859 | 0.425 | 0.025 | 0.4 | 4.44E-153 | 4 |
| Aurka | 3.95E-154 | 0.521977 | 0.385 | 0.018 | 0.367 | 1.01E-149 | 4 |
| Kif2c | 1.64E-152 | 0.566692 | 0.445 | 0.031 | 0.414 | 4.21E-148 | 4 |
| Foxm1 | 3.38E-152 | 0.618715 | 0.425 | 0.027 | 0.398 | 8.67E-148 | 4 |
| Mad2l1 | 2.99E-151 | 0.551931 | 0.495 | 0.042 | 0.453 | 7.69E-147 | 4 |
| Stmn1 | 8.17E-151 | 1.248314 | 0.89 | 0.254 | 0.636 | 2.10E-146 | 4 |
| Cks2 | 1.56E-145 | 0.681952 | 0.518 | 0.053 | 0.465 | 4.01E-141 | 4 |
| Ube2c | 1.84E-143 | 1.332158 | 0.642 | 0.107 | 0.535 | 4.71E-139 | 4 |
| Clspn | 9.01E-142 | 0.527748 | 0.344 | 0.015 | 0.329 | 2.31E-137 | 4 |
| Fbxo5 | 1.86E-141 | 0.641905 | 0.458 | 0.039 | 0.419 | 4.78E-137 | 4 |
| Slc43a3 | 7.80E-141 | 0.628208 | 0.495 | 0.048 | 0.447 | 2.00E-136 | 4 |
| Dbf4 | 3.58E-138 | 0.59598 | 0.505 | 0.052 | 0.453 | 9.18E-134 | 4 |
| Diaph3 | 8.65E-137 | 0.597403 | 0.448 | 0.038 | 0.41 | 2.22E-132 | 4 |
| Pimreg | 8.55E-136 | 0.517867 | 0.341 | 0.017 | 0.324 | 2.19E-131 | 4 |
| Knstrn | 1.11E-135 | 0.707535 | 0.458 | 0.043 | 0.415 | 2.86E-131 | 4 |
| Tmpo | 6.01E-131 | 1.04631 | 0.846 | 0.23 | 0.616 | 1.54E-126 | 4 |
| Mcm5 | 2.88E-127 | 0.705452 | 0.538 | 0.071 | 0.467 | 7.40E-123 | 4 |
| Atad2 | 7.85E-126 | 0.8638 | 0.559 | 0.08 | 0.479 | 2.01E-121 | 4 |
| Lmnb1 | 2.00E-125 | 0.86921 | 0.679 | 0.129 | 0.55 | 5.14E-121 | 4 |
| Dnajc9 | 1.13E-123 | 0.638549 | 0.565 | 0.08 | 0.485 | 2.89E-119 | 4 |
| Lig1 | 9.54E-122 | 0.935988 | 0.619 | 0.108 | 0.511 | 2.45E-117 | 4 |
| Cdkn3 | 1.29E-119 | 0.638004 | 0.435 | 0.045 | 0.39 | 3.32E-115 | 4 |
| Tuba1c | 1.18E-115 | 1.133272 | 0.843 | 0.263 | 0.58 | 3.04E-111 | 4 |
| Cbx5 | 8.47E-111 | 0.851774 | 0.746 | 0.179 | 0.567 | 2.17E-106 | 4 |
| Tyms | 1.04E-110 | 0.558873 | 0.495 | 0.068 | 0.427 | 2.67E-106 | 4 |
| Rangap1 | 5.17E-110 | 0.900286 | 0.763 | 0.19 | 0.573 | 1.33E-105 | 4 |
| Arhgap11a | 1.20E-101 | 0.518637 | 0.455 | 0.06 | 0.395 | 3.09E-97 | 4 |
| Tubb4b | 6.48E-101 | 0.598785 | 0.532 | 0.089 | 0.443 | 1.66E-96 | 4 |
| Pmf1 | 7.81E-100 | 0.541356 | 0.559 | 0.099 | 0.46 | 2.01E-95 | 4 |
| Ccdc34 | 1.28E-98 | 0.552423 | 0.525 | 0.086 | 0.439 | 3.28E-94 | 4 |
| Hmgn2 | 9.24E-97 | 0.557395 | 0.609 | 0.12 | 0.489 | 2.37E-92 | 4 |
| Ncaph | 2.54E-91 | 0.56819 | 0.518 | 0.092 | 0.426 | 6.51E-87 | 4 |
| H2afz | 2.22E-90 | 0.682832 | 0.706 | 0.185 | 0.521 | 5.69E-86 | 4 |
| Nucks1 | 6.57E-90 | 0.853772 | 0.799 | 0.256 | 0.543 | 1.69E-85 | 4 |
| Pcna | 4.94E-88 | 1.001826 | 0.736 | 0.233 | 0.503 | 1.27E-83 | 4 |
| Iqgap3 | 5.71E-87 | 0.550499 | 0.512 | 0.094 | 0.418 | 1.47E-82 | 4 |
| Timeless | 7.75E-87 | 0.50511 | 0.334 | 0.037 | 0.297 | 1.99E-82 | 4 |
| Cdkn2d | 1.62E-84 | 0.556901 | 0.455 | 0.077 | 0.378 | 4.15E-80 | 4 |
| Mcm6 | 6.78E-84 | 0.606381 | 0.465 | 0.081 | 0.384 | 1.74E-79 | 4 |
| Reep4 | 7.83E-84 | 0.765889 | 0.659 | 0.174 | 0.485 | 2.01E-79 | 4 |
| Sae1 | 1.82E-79 | 0.601752 | 0.625 | 0.158 | 0.467 | 4.67E-75 | 4 |
| Gas2l3 | 2.12E-79 | 0.560657 | 0.371 | 0.053 | 0.318 | 5.44E-75 | 4 |
| H2afv | 2.30E-78 | 0.749139 | 0.763 | 0.243 | 0.52 | 5.91E-74 | 4 |
| Usp1 | 3.29E-76 | 0.630077 | 0.629 | 0.166 | 0.463 | 8.46E-72 | 4 |
| Ranbp1 | 9.29E-74 | 0.641769 | 0.736 | 0.233 | 0.503 | 2.39E-69 | 4 |
| Selenoh | 2.41E-73 | 0.567621 | 0.669 | 0.187 | 0.482 | 6.17E-69 | 4 |
| Hjurp | 5.07E-71 | 0.809654 | 0.699 | 0.228 | 0.471 | 1.30E-66 | 4 |
| Hat1 | 5.42E-67 | 0.502228 | 0.525 | 0.126 | 0.399 | 1.39E-62 | 4 |
| Gm4617 | 2.00E-66 | 0.668642 | 0.783 | 0.288 | 0.495 | 5.12E-62 | 4 |
| Mcm3 | 4.65E-63 | 0.592122 | 0.542 | 0.142 | 0.4 | 1.19E-58 | 4 |
| Ckap5 | 5.61E-58 | 0.57245 | 0.679 | 0.231 | 0.448 | 1.44E-53 | 4 |
| Gm42047 | 2.23E-57 | 0.519381 | 0.288 | 0.043 | 0.245 | 5.72E-53 | 4 |
| Gm4204 | 2.60E-52 | 0.599935 | 0.716 | 0.27 | 0.446 | 6.67E-48 | 4 |
| Emp3 | 1.08E-51 | 0.543333 | 0.716 | 0.272 | 0.444 | 2.78E-47 | 4 |
| Msr1 | 2.73E-49 | 0.645734 | 0.548 | 0.174 | 0.374 | 7.01E-45 | 4 |
| Anp32e | 1.61E-46 | 0.594984 | 0.712 | 0.293 | 0.419 | 4.14E-42 | 4 |
| Plin2 | 1.29E-42 | 0.561153 | 0.679 | 0.28 | 0.399 | 3.32E-38 | 4 |
| Capg | 3.15E-42 | 0.510891 | 0.712 | 0.295 | 0.417 | 8.08E-38 | 4 |
| Fn1 | 2.30E-41 | 0.794814 | 0.592 | 0.228 | 0.364 | 5.90E-37 | 4 |
| Oasl2 | 8.84E-63 | 0.939994 | 0.631 | 0.228 | 0.403 | 2.27E-58 | 5 |
| Slfn5 | 3.95E-58 | 0.900126 | 0.57 | 0.195 | 0.375 | 1.01E-53 | 5 |
| Stat2 | 2.72E-51 | 0.841512 | 0.654 | 0.281 | 0.373 | 6.99E-47 | 5 |
| Ifit2 | 1.55E-50 | 0.819 | 0.326 | 0.069 | 0.257 | 3.98E-46 | 5 |
| Rnf213 | 4.76E-50 | 0.760753 | 0.671 | 0.299 | 0.372 | 1.22E-45 | 5 |
| Oas2 | 8.01E-50 | 0.51075 | 0.279 | 0.049 | 0.23 | 2.06E-45 | 5 |
| Ifi204 | 6.70E-46 | 0.793477 | 0.55 | 0.221 | 0.329 | 1.72E-41 | 5 |
| Isg15 | 3.41E-40 | 0.71172 | 0.376 | 0.113 | 0.263 | 8.76E-36 | 5 |
| Ifit3 | 3.33E-39 | 1.043507 | 0.453 | 0.165 | 0.288 | 8.54E-35 | 5 |
| Irf7 | 1.45E-38 | 0.671152 | 0.419 | 0.143 | 0.276 | 3.73E-34 | 5 |
| Ifi209 | 5.10E-37 | 0.546332 | 0.372 | 0.111 | 0.261 | 1.31E-32 | 5 |
| Phf11b | 3.99E-31 | 0.580746 | 0.51 | 0.227 | 0.283 | 1.03E-26 | 5 |
| Stat1 | 5.21E-27 | 0.534468 | 0.55 | 0.284 | 0.266 | 1.34E-22 | 5 |
| Tor3a | 2.77E-24 | 0.57833 | 0.527 | 0.276 | 0.251 | 7.12E-20 | 5 |
| C4b | 6.00E-23 | 0.508009 | 0.376 | 0.156 | 0.22 | 1.54E-18 | 5 |
| Ch25h | 4.81E-129 | 1.264233 | 0.688 | 0.105 | 0.583 | 1.23E-124 | 6 |
| Igf1 | 1.41E-127 | 1.066369 | 0.602 | 0.078 | 0.524 | 3.62E-123 | 6 |
| Rab7b | 8.11E-119 | 0.813077 | 0.662 | 0.104 | 0.558 | 2.08E-114 | 6 |
| Htr2b | 5.10E-108 | 0.525707 | 0.407 | 0.036 | 0.371 | 1.31E-103 | 6 |
| Adam8 | 2.22E-103 | 0.61154 | 0.437 | 0.046 | 0.391 | 5.71E-99 | 6 |
| Cd5l | 3.48E-103 | 0.995002 | 0.468 | 0.055 | 0.413 | 8.93E-99 | 6 |
| Msr1 | 3.53E-103 | 1.141051 | 0.749 | 0.167 | 0.582 | 9.05E-99 | 6 |
| Lpl | 1.08E-99 | 1.423388 | 0.827 | 0.23 | 0.597 | 2.78E-95 | 6 |
| Igf2r | 1.09E-98 | 0.519147 | 0.433 | 0.047 | 0.386 | 2.79E-94 | 6 |
| Gla | 3.50E-98 | 0.664688 | 0.636 | 0.114 | 0.522 | 8.99E-94 | 6 |
| Lilr4b | 1.21E-96 | 0.641577 | 0.476 | 0.061 | 0.415 | 3.11E-92 | 6 |
| Lgals3 | 6.09E-87 | 1.40713 | 0.835 | 0.266 | 0.569 | 1.56E-82 | 6 |
| Nceh1 | 1.86E-86 | 0.578334 | 0.61 | 0.115 | 0.495 | 4.78E-82 | 6 |
| Myo1e | 9.52E-86 | 0.743182 | 0.779 | 0.2 | 0.579 | 2.44E-81 | 6 |
| Plin2 | 4.07E-83 | 1.090859 | 0.835 | 0.277 | 0.558 | 1.04E-78 | 6 |
| Capg | 5.64E-74 | 0.853313 | 0.84 | 0.294 | 0.546 | 1.45E-69 | 6 |
| Ell2 | 5.20E-73 | 0.570453 | 0.576 | 0.119 | 0.457 | 1.33E-68 | 6 |
| Ifi207 | 1.13E-72 | 0.591663 | 0.628 | 0.14 | 0.488 | 2.89E-68 | 6 |
| Ccl4 | 3.83E-70 | 1.343645 | 0.623 | 0.153 | 0.47 | 9.83E-66 | 6 |
| Tmem106a | 4.34E-70 | 0.586519 | 0.68 | 0.169 | 0.511 | 1.11E-65 | 6 |
| Itga5 | 1.09E-69 | 0.740948 | 0.775 | 0.226 | 0.549 | 2.80E-65 | 6 |
| Emp3 | 1.22E-65 | 0.709199 | 0.827 | 0.274 | 0.553 | 3.13E-61 | 6 |
| Ccl3 | 1.28E-64 | 1.044315 | 0.654 | 0.174 | 0.48 | 3.30E-60 | 6 |
| Iqgap1 | 5.89E-62 | 0.724821 | 0.827 | 0.291 | 0.536 | 1.51E-57 | 6 |
| Csf1 | 8.43E-62 | 0.766716 | 0.727 | 0.221 | 0.506 | 2.16E-57 | 6 |
| Rap2b | 1.53E-60 | 0.572364 | 0.719 | 0.213 | 0.506 | 3.93E-56 | 6 |
| Fabp5 | 3.98E-60 | 0.716432 | 0.554 | 0.137 | 0.417 | 1.02E-55 | 6 |
| Anxa2 | 2.83E-59 | 0.507003 | 0.641 | 0.174 | 0.467 | 7.25E-55 | 6 |
| Ms4a7 | 7.28E-58 | 0.521651 | 0.411 | 0.077 | 0.334 | 1.87E-53 | 6 |
| Atf3 | 1.10E-57 | 0.526199 | 0.407 | 0.075 | 0.332 | 2.81E-53 | 6 |
| Nrp2 | 1.54E-56 | 0.600806 | 0.71 | 0.221 | 0.489 | 3.95E-52 | 6 |
| Mpp1 | 1.48E-55 | 0.501573 | 0.675 | 0.197 | 0.478 | 3.81E-51 | 6 |
| Serpinb6a | 1.35E-54 | 0.545424 | 0.524 | 0.13 | 0.394 | 3.46E-50 | 6 |
| Hmox1 | 2.87E-53 | 0.642733 | 0.758 | 0.253 | 0.505 | 7.36E-49 | 6 |
| Cst7 | 1.96E-51 | 0.567917 | 0.719 | 0.236 | 0.483 | 5.04E-47 | 6 |
| Clec7a | 3.26E-51 | 0.696031 | 0.545 | 0.15 | 0.395 | 8.36E-47 | 6 |
| Npl | 3.69E-46 | 0.549082 | 0.645 | 0.215 | 0.43 | 9.48E-42 | 6 |
| Vat1 | 6.64E-46 | 0.533539 | 0.571 | 0.173 | 0.398 | 1.70E-41 | 6 |
| Tlr2 | 1.32E-40 | 0.566343 | 0.771 | 0.297 | 0.474 | 3.38E-36 | 6 |
| Nes | 7.53E-35 | 0.54017 | 0.355 | 0.087 | 0.268 | 1.93E-30 | 6 |
| Slc15a3 | 1.96E-32 | 0.510476 | 0.719 | 0.295 | 0.424 | 5.03E-28 | 6 |
| Fn1 | 9.24E-28 | 0.574625 | 0.597 | 0.236 | 0.361 | 2.37E-23 | 6 |

| **Table S5：DAM, IRM, ATM and senescence associated gene lists** | | | |
| --- | --- | --- | --- |
| Disease associated Microglia (DAM) | Injury Responsive Microglia (IRM) | Axon Tract Microglia (ATM) | Senescence associated gene (SASP) |
|  |  |  |  |
| Itgax | Apoe | Spp1 | Cdkn2a |
| Cst7 | Ifi27l2a | Gpnmb | Bmi1 |
| Fam20c | Ifitm3 | Igf1 | Trp53 |
| Ccl4 | Ccl12 | Lgals3 | Hmga1 |
| Csf1 | Ccl4 | Cd9 | Chek1 |
| 5430435G22Rik | Lgals3bp | Fabp5 | Chek2 |
| Ccl3 | Cst7 | Lpl | Prodh |
| Clec7a | Cd52 | Syngr1 | Tnfrsf10b |
| Baiap2l2 | Bst2 | Pld3 | Cdkn1a |
| Lpl | H2-D1 | Ctsl | Dao |
| Ank | Lpl | Lgals1 | Ccl2 |
| Cox6a2 | Ccl3 | Lilrb4a | Ccl24 |
| Spp1 | Ifit3 | Ccl9 | Ccl3 |
| Axl | Isg15 | Anxa5 | Ccl5 |
| Igf1 | Ifi204 | Gm1673 | Ctnnb1 |
| Psat1 | H2-K1 | Csf1 | Cxcl1 |
| Capg | Irf7 | Cd63 | Cxcl10 |
| Lyz2 | Cxcl10 | Gm10116 | Cxcl12 |
| Apoe | Oasl2 | Anxa2 | Cxcl2 |
| Lgi2 | Rtp4 | Apoe | Cxcl16 |
| Nceh1 | Ccl5 | Lag3 | Hgf |
| H2-D1 | B2m | Ctsb | Hmgb1 |
| Cd83 | Lyz2 | S100a1 | Icam1 |
| Fxyd5 | Rps28 | Ccl6 | Igfbp2 |
| Apbb2 | Rps29 | Fabp3 | Igfbp3 |
| Dpp7 | Rpl41 | Lyz2 | Igfbp4 |
| Ifi27l2b | Ctsb | Gpx3 | Igfbp5 |
| Cd63-ps | Spp1 | Plin2 | Igfbp6 |
| Mif | Ch25h | Cstb | Igfbp7 |
| Cadm1 | Gm10076 | Plaur | Il15 |
| Ccl6 | Slfn2 | Folr2 | Il18 |
| Sulf2 | H2-Q7 | Aplp2 | Il1a |
| Cd52 | Zfos1 | Ftl1 | Il1b |
| Cd63 | Tspo | Vat1 | Il2 |
| Kcnj2 | Stat1 | Gng12 | Il6 |
| Myo1e | Cd72 | Mif | Mif |
| Scpep1 | Rpl32 | Pkm | Mmp12 |
| Ramp1 | Rpl10a | Hpse | Mmp13 |
| Aplp2 | Rpl37a | Fam20c | Mmp14 |
| Rps24-ps3 | Usp18 | Ldha | Pgf |
| Lgals3bp | Ccl2 | Atp6v0d2 | Plat |
| Cd34 | Rpl38 | Tmem106a | Timp2 |
| Slamf9 | Axl | Gpr137b | Serpine1 |
| H2-K1 | Cd63 | Apoc1 | Ccl3 |
| Crlf2 | Tor3a | Tpi1 | Ccl4 |
| Ftl1 | Rps19 | Ctsz | Ang |
| Gnas | Rpl36 | Lamp1 | Csf2 |
| AL928935.1 | Phf11b | Ccl3 | Kitl |
| Bcl2a1b | Rpl35 | Ifitm2 | Serpine2 |
| Gm6030 | Rpl39 | Sepw1 | Tnfrsf1a |
| Cd9 | Rpl23 | Slpi | Hgf |
| Ctsb | Mif | Adssl1 | Nrg1 |
| Cd74 | Rps16 | Cd68 | Ereg |
| Serpine2 | Oas1a | Aig1 | Areg |
| Cxcl16 | Uba52 | Ybx1 |  |
| Gm16247 | Ifit2 | Dpp7 |  |
| Gusb | Zbp1 | Slc16a3 |  |
| Echs1 | Rps26 | Ctsa |  |
| Pld3 | Rsad2 | Bnip3 |  |
| Gm7331 | Cd83 | Npc2 |  |
| Gm7363 | Gm4951 | Pgk1 |  |
| Gm5239 | Rpl37 | Sepp1 |  |
| Rpl36a-ps1 | Trim30a | Timp2 |  |
| Ctsz | Rps18 | Sh3bgrl |  |
| Syngr1 | Sp100 | Eno1b |  |
| Scd2 | Rps20 | Itgax |  |
| Npc2 | Ly6e | Crip1 |  |
| Gm5805 | Rps24 | Creg1 |  |
| Gm10136 | Fau | Psmb6 |  |
| Gm9493 | Fcgr4 | Gatm |  |
| Gm15772 | Rps12 | Aprt |  |
| Sdc3 | Ly6a | X0610012G03Rik |  |
| Gm10186 | Parp14 | Uap1l1 |  |
| Tyrobp | Rpl19 | Gnas |  |
| Mir682 | Rps15a | Chchd10 |  |
| Naglu | Rps27a | Ank |  |
| Gm13192 | Ifit1 | Plek |  |
| Mt1 | Rpl24 | Uba52 |  |
| Rpl23 | Grn | Npl |  |
| Gm10443 | Rpsa | Ctsd |  |
| Ssr4 | Rps27rt | Atp1a1 |  |
| Gm13826 | Npc2 | Eno1 |  |
| Atp6v0c | Gm9843 | Dab2 |  |
| Gm10279 | Rps27 | Ramp1 |  |
| Gm5963 | Xaf1 | Clta |  |
| Fth1 | Rpl12 | Wbp5 |  |
| Gm5244 | Rpl26 | Gpi1 |  |
| Hif1a | Rps2 | Cyba |  |

Table S6-1： Ch25h expression in total cells and microglia

|  | Cell number (sham) | *Ch25h* average expression (sham) | Cell number (stroke) | *Ch25h* average expression (stroke) | log2(fold change)  (stroke vs sham) | p-value |
| --- | --- | --- | --- | --- | --- | --- |
| Total cells | 5517 | 0.0101 | 5709 | 0.5417 | 0.2243 | 5.2963e-147 |
| Total MG | 870 | 0.0087 | 2167 | 1.0047 | 0.3793 | 7.6768e-47 |

Table S6-2： Ch25h expression in microglia subclusters

|  | Cell numbers | *Ch25h* average expression | pct.1 | pct.2 | log2(fold change) | p-value |
| --- | --- | --- | --- | --- | --- | --- |
| MG0 | 890 | 0.0247 | 0.007 | 0.209 | -0.9700 | 1.0223e-45 |
| MG1 | 597 | 0.2534 | 0.111 | 0.159 | -0.5486 | 9.1724e-4 |
| MG2 | 391 | 0.2021 | 0.051 | 0.164 | -0.5791 | 5.253e-9 |
| MG3 | 331 | 0.7853 | 0.097 | 0.156 | 0.0610 | 0.0131 |
| MG4 | 299 | 1.4952 | 0.398 | 0.123 | 0.6101 | 3.4286e-35 |
| MG5 | 298 | 0.9328 | 0.178 | 0.147 | 0.1884 | 0.1670 |
| MG6 | 231 | 4.1015 | 0.688 | 0.105 | 1.8239 | 1.234e-129 |

| **Table S7：List of interferon response gene**s | | | | | |
| --- | --- | --- | --- | --- | --- |
| Interferon response genes | | | | | |
| Ifit3 | Ifitm3 | Usp18 | Ifi211 | Ifi204 | Irf7 |
| Ifit2 | Ifi206 | Oasl2 | Ifit3b | Tgtp2 | Ifi208 |
| Ifit1 | Iigp1 | Oas1a | Ifi207 | Rsad2 | Rtp4 |
| Oasl1 | Oas2 | RP24-84E18.1 | Ccl12 | Ifi213 | Zbp1 |
| Phf11b | Slfn5 | Mx1 | Ifi47 | Ifi209 | Gm4951 |
| Oas1g | Cxcl10 | Cmpk2 | Phf11d | Stat1 | Oas3 |
| Gbp6 | Sp100 | Mx2 | Slfn2 | Nlrc5 | Ms4a4c |
| Stat2 | Rnf213 | Phf11a | Bst2 | Ccl2 | Ifi27l2a |
| Gm12250 | Pml | Fgl2 | Xaf1 | Isg20 | Irgm1 |
| Tor3a | Parp14 | Dhx58 | Gm7609 | Ifih1 | Helz2 |
| Ly6e | Igtp | F830016B08Rik | Ddx58 | Trim30a | Gbp4 |
| Gm7592 | Gm4841 | Gbp2 | Ly6a | Tgtp1 | Gm6904 |
| BC147527 | Socs1 | Eif2ak2 | Fcgr1 | Slfn9 | Gm45418 |
| H2-T23 | Slfn8 | Sdc3 | Gm20559 | Trim30d | Ube2l6 |
| Batf2 | Ccl5 | Ifi44 | Samd9l | Oas1b | Gm1966 |
| H2-Q6 | Gm5431 | B430306N03Rik | Parp9 | Gm8995 | Gm15433 |
| Adar | H2-Q7 | Tnfsf10 | Znfx1 | Ifi35 | Parp12 |
| Herc6 | Trim30c | Irf1 | Scimp | Nampt | Gm5970 |
| Trafd1 | Gbp3 | Ifi214 | C4b | Phf11c | Clic4 |
| Slc25a22 | H2-T24 | Lgals9 | Samhd1 | Trim30b | Ly6i |
| Fcgr4 | Daxx | Siglec1 | Zufsp | Gbp9 | Tpst1 |
| Gm44935 | Dck | Parp10 | Psme1 | Ddx60 | Trim25 |
| Prr5l | Vcam1 | Gbp5 | Ogfr | Zc3hav1 | Epsti1 |
| Gpr84 | Mthfr | Tap1 | Treml2 | Slamf8 | Hap1 |
| Ccdc86 | 9330175E14Rik | Nmi | Pik3ap1 | 1600014C10Rik | Etnk1 |
| Gm17435 | Tor1aip1 | Gbp7 | Shisa5 | Timeless | Psmb9 |
| Gm2065 | Irf9 | Chic1 | Mybph | Tlr3 | Gm20429 |
| Apobec3 | Zfas1 | Tctn2 | Trim12c | Dtx3l | Naa25 |
| Setdb2 | Gm12216 | 2310001H17Rik | Sap30 | Tapbp | Arid5a |
