# Supplementary material for: Novel CH25H+ and OASL+ microglia subclusters play distinct roles in cerebral ischemic stroke
Source: J Neuroinflammation. 2023 May 15;20:115. doi: 10.1186/s12974-023-02799-6 (PMC10184422; doi:10.1186/s12974-023-02799-6)
Supplement: Supplementary file 2 — Additional file 2. Additional figures and methods. [file 12974_2023_2799_MOESM2_ESM.docx]

**Additional file 2: Figures and Methods**

**Novel CH25H^+^ and OASL^+^ Microglia Subclusters Play Distinct Roles in Cerebral Ischemic Stroke**

Yueman Zhang^#1^, Yunlu Guo^#1^, Ruqi Li^2^, Tingting Huang^1^, Yan Li^1^, Wanqin Xie^1^, Chen Chen^1^, Weijie Chen^1^, Jieqing Wan^2^, Weifeng Yu^1^, Peiying Li*^1,3^

^1^ Department of Anesthesiology, Key Laboratory of the Ministry of Education, Renji Hospital, Shanghai Jiao Tong University School of Medicine, Shanghai, China

^2^Department of Neurological Surgery, Renji Hospital, Shanghai Jiao Tong University School of Medicine, Shanghai, China

^3^Clinical Research Center, Renji Hospital, Shanghai Jiao Tong University School of Medicine, Shanghai, China

Running Title: Ischemic stroke associated microglia

*Correspondence:

Dr. Peiying Li, MD, PhD.

Department of Anesthesiology, Clinical Research Center

Renji Hospital, Shanghai Jiao Tong University School of Medicine

Shanghai, China; 160 Pujian Rd

Shanghai 200127, China

Email: peiyingli.md@gmail.com

Phone: 8615800616866

#These authors contributed equally to this work.

**Methods**

**Cerebral Ischemia Model**

Mice were anesthetized with 3% isoflurane in 67%:30% N2O/ O2 (induction), until they were unresponsive to the tail pinch test and were then fitted with a nose cone providing 1.5% isoflurane for anesthesia maintenance. Transient middle cerebral artery occlusion (tMCAO) was induced by placing a monofilament in the common carotid artery, details can be found in supplementary material. advancing it to the origin of the middle cerebral artery, and left in position for 60 minutes until reperfusion. Body temperature was maintained at 37 ± 0.5°C with a heating pad during surgical procedures. Animals were excluded from further experiments if they did not show a cerebral blood flow (CBF) reduction of at least 75%. Distal middle cerebral artery occlusion (dMCAO) was induced as described below. Briefly, mice were anesthetized with 2% isoflurane in a 30% O2/68% N2O mixture under spontaneous breathing conditions. A skin incision was made at the neck and the left common carotid artery was exposed and ligated. After the neck incision was sutured, another skin incision was made between the left eye and ear. The temporal muscle was dissected and a burr hole was opened to expose the distal part of middle cerebral artery occlusion (MCA). The dura mater was then cut and the distal MCA was coagulated with low‐intensity bipolar electrocautery (Shanghai Hutong Electronics Co. Ltd.) at the immediate lateral part of the rhinal fissure. Sham‐operated animals underwent anesthesia and surgical exposure of arteries but without artery occlusion. Sham-operated animals underwent the same anesthesia and surgical procedures except tMCAO/dMCAO.

**Study timeline**

The overall study timeline is as follows:

1) 6 hours, Day 1, Day3, and Day5 after tMCAO: Collect tissue for immunostaining.

2) Day 3 after tMCAO: Collect tissue for PCR, immunostaining, flow cytometry, scRNA-sequencing.

3) Day 3 after tMCAO: Collect tissue for immunostaining.

4) Day 1 to Day 28: Day 1, 3, 5, 7, 14, 28 for modified Garcia Score, grid walk, and Rotarod test; Day 23 to 28 for Morris water maze test.

We used a total of 80 mice in this study. Specifically, 6 mice underwent MCAO surgery and were included in the scRNA-seq analysis (3 for tMCAO group and 3 for sham group). For the study of Ch25h, we used a total of 20 mice (10 for Ch25h^+/-^ and 10 for Ch25h^-/-^) for knockout efficiency test, infarct volume and neuroinflammation assessment. For the behavioral tests, we used 8 mice per group (total 32 mice). For the aged mice, 6 mice underwent dMCAO surgery and 6 for sham group.

**MAP2 and IgG staining**

Infarct volume was evaluated by microtubule-associated protein 2 (MAP-2) immunofluorescence staining. BBB leakage was evaluated by extravasation of plasma IgG. We incubated the brain sections with primary antibodies against anti-MAP2（Rabbit monoclonal), followed by incubation with fluorescent secondary antibody Anti-Rabbit IgG H&L (Alexa Fluor® 488) and Anti-Mouse IgG H&L (Alexa Fluor® 594). The sections were then imaged using a confocal microscope, and the infarct volume or IgG^+^ area was determined with NIH Image J (1.52a) analysis by an investigator who was blinded to the experimental group assignment.

**qPCR**

The sham brain and ischemic brain tissues were grinded to homogenate in TRIzol reagent (Vazyme, R401-01). The total RNA was isolated using tissue RNA purification kit (EZBioscience, RN001A). cDNA was synthesized using a color reverse transcription kit (EZBioscience, A0010CGQ). qPCR was performed using 2x color SYBR Green qPCR Master Mix (EZBioscience, A0012-R2) in CFX384 Real-Time PCR System (BioRad, 1-855-484). All reagents were used according to manufacturer’s instructions.

The following mouse gene primer sequences were used:

*Ch25h*: ATGGGCTGCTACAACGGTTC (Fwd); CCTTGTCCTTATGGTGTCCCAG (Rev)

*β-actin*: GGCTGTATTCCCCTCCATCG (Fwd); CCAGTTGGTAACAATGCCATGT (Rev)

**Behavioral tests**

Sensorimotor functions was assessed by the modified Garcia Score^1^, grid walk^2^, and Rotarod test ^3^, which were performed as previously described to assess before and after surgery by investigators who were blinded to experimental group assignments. The modified Garcia Score is a well‐established sensorimotor assessment system consisting of five individual tests, of which one measures sensory function while four measure motor function. We scored each test from 0 to 3 (maximal score = 15): (a) body proprioception, (b) forelimb walking, (c) limb symmetry, (d) lateral turning, and (e) climbing as described. The grid walk test was performed according to Rogers et al. ^2^ with slight modifications. In the grid walk test, mice were placed on an elevated steel grid and the foot fault errors (when the animal’s forelimb was misplaced and fell through the grid) were recorded as the mice moved. Data are presented as percentage of foot fault errors for the right impaired forelimb relative to the total amount of right forelimb steps. For the rotarod test, mice were forced to run on a rotating rod (YLS-4D) with speeds starting at 4 rpm and accelerating to 40 rpm within 300 seconds. Three consecutive trials were conducted for each mouse, with an interval of 15 minutes. The time at which a mouse fell off the rod was recorded as the latency to fall. Data were expressed as mean values from three trials per day.

Cognitive function was analyzed using the Morris water maze test, as described previously^4^. A square platform (11 × 11 cm^2^) was submerged 2 cm beneath the water surface in a circular pool (diameter = 10^9^ cm) filled with opaque water. Mice were placed into the pool from one of the four locations and allowed to locate the hidden platform for 60s. Each mouse was trained on 3 trials (with randomly assigned starting positions) per day to locate the platform for three consecutive days before tMCAO. At the end of each trial, the mouse was placed on the platform or allowed to stay on the platform for 30s with prominent spatial cues displayed around the room. Trials were recorded with Anymaze system (Stoelting, US). In the learning test, three trials were performed on each day. The time spent to reach the platform was recorded to reflect spatial learning. In the memory test, the platform was removed and a single 60s probe trial was conducted. Time spent in the goal quadrant (where the platform was previously located) was recorded to reflect spatial memory.

**Figures and Legends**

**Figure S1**


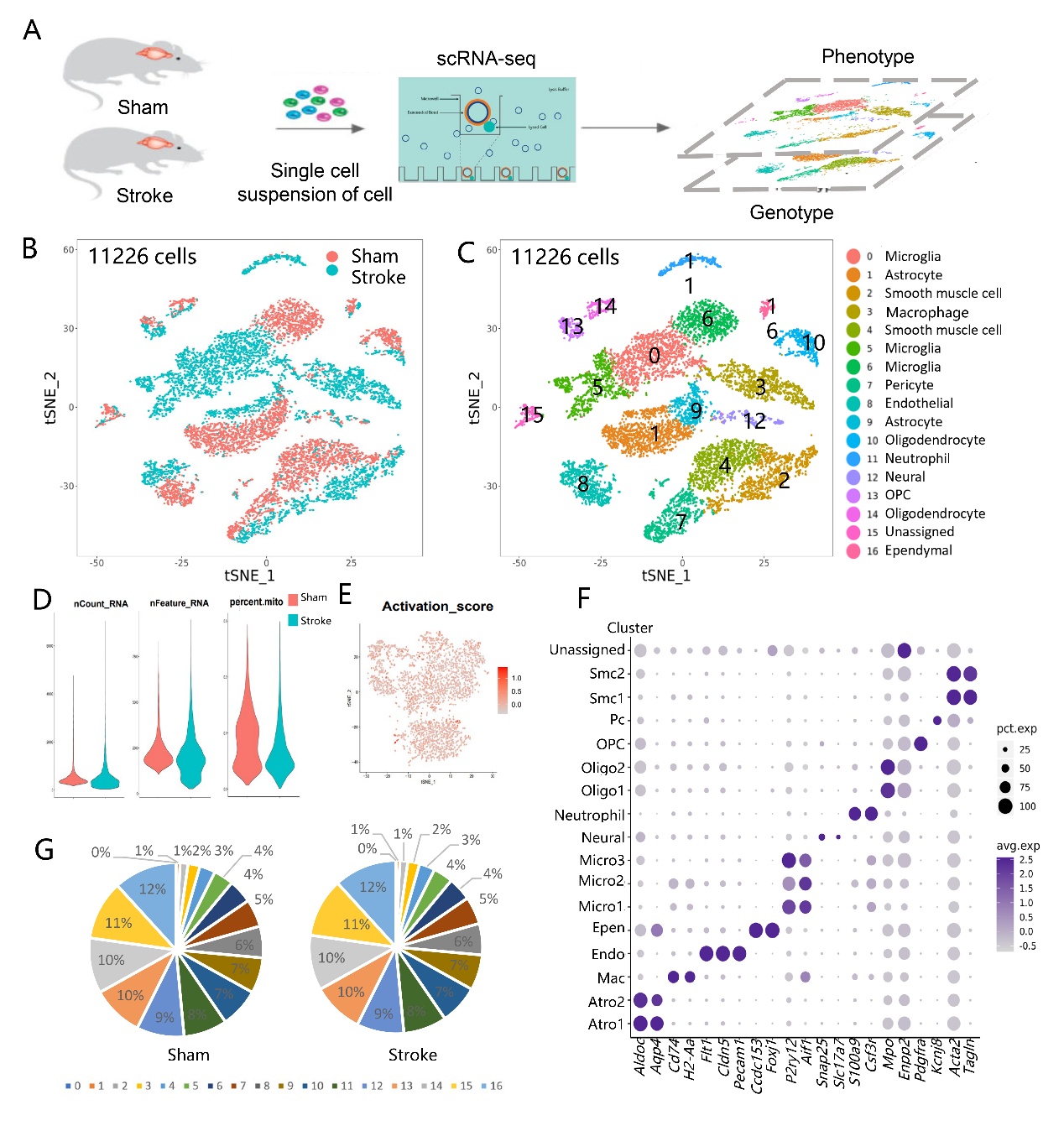


**Figure S1** **Sixteen Distinct Cell Subpopulations Identified in the Ischemic Mice Brain**

(A) Diagram of experimental design. All cells were sequenced with a modified BD Rhapsody scRNA-seq technique.

(B)t-SNE representation of 11226 cells from 3 sham (n=5517 cells) and 3 tMCAO (n=5709 cells) mice brain. Dots, individual cells; colors, different origin.

(C) t-SNE representation of the 11226 single cells shows the cellular heterogeneity of brain cells. Dots, individual cells; colors, cell clusters.

(D) nGene, nUMI and percent of mitochondrial of 11226 individual brain cells from 3 sham (n=5517 cells) and 3 tMCAO (n=5709 cells) mice.

(E) Activation score of microglia from 3 sham and 3 tMCAO mice.

(F) Marker genes in each brain cluster. The size of the dot indicates the percentage of cells expressing the gene in each cluster, and the color depicts the average normalized transcript counts in the cells.

(G) The pie chart shows the percentage of each cluster in (b) in different groups (sham & stroke).

**Figure S2**


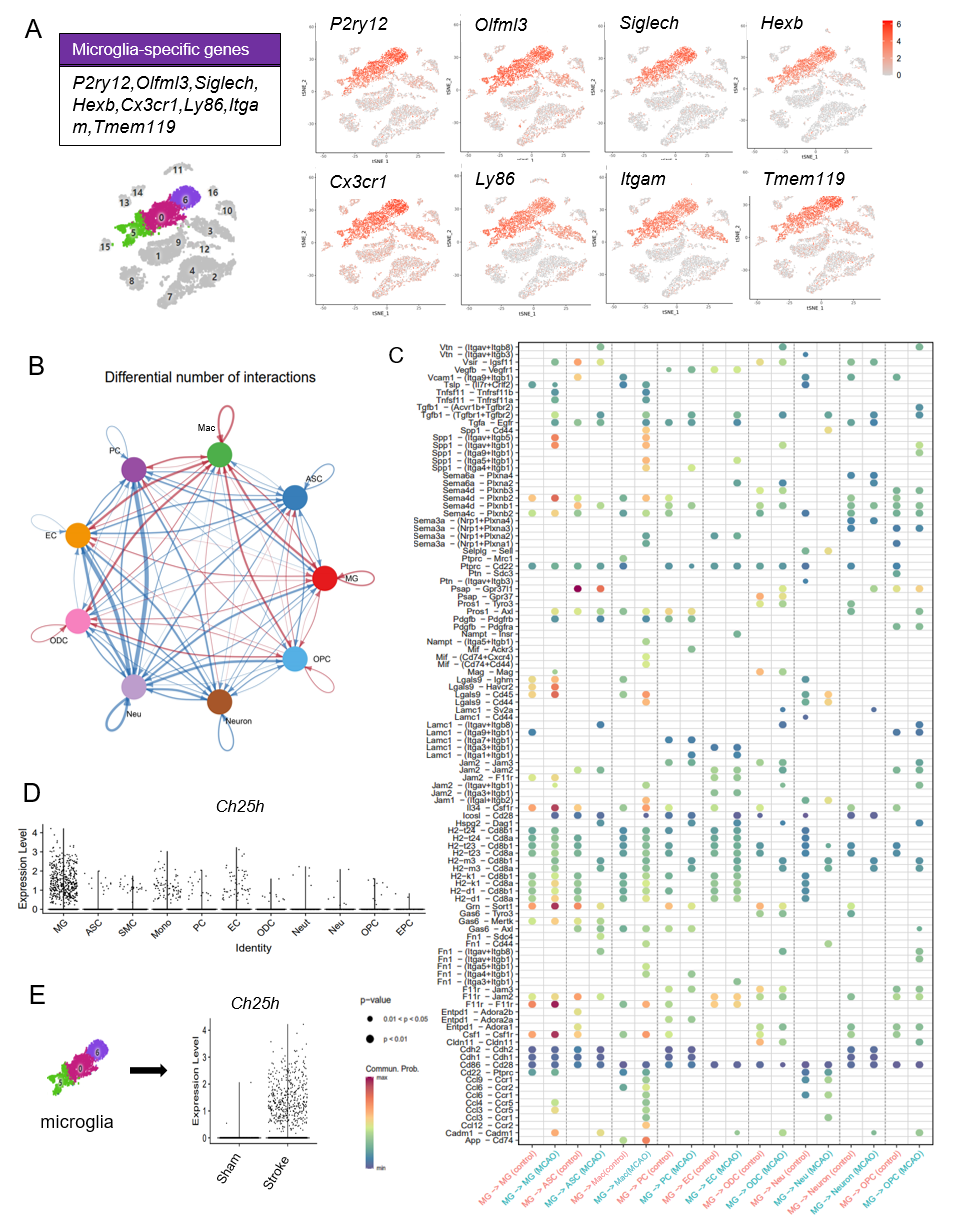


**Figure S2 Interactions of microglia with other brain cells after ischemic stroke**

(A) Projection of key marker genes onto the graph plot of microglia. The color represents the Log2 normalized transcript counts.

(B) CellChat reveals cell communications among each cell type, with SMC and ependymal cells excluded.

(C) The dot plot representing all ligand–receptor interactions between microglia and each cell type, respectively. The color gradient indicates the communication probability, the size indicates p-values.

(D) Violin plots of Ch25h from sham and tMCAO mice brain. Dots, individual cells.

(E) Violin plots of Ch25h of microglia from sham and tMCAO mice brain. Dots, individual cells.

**Figure S3
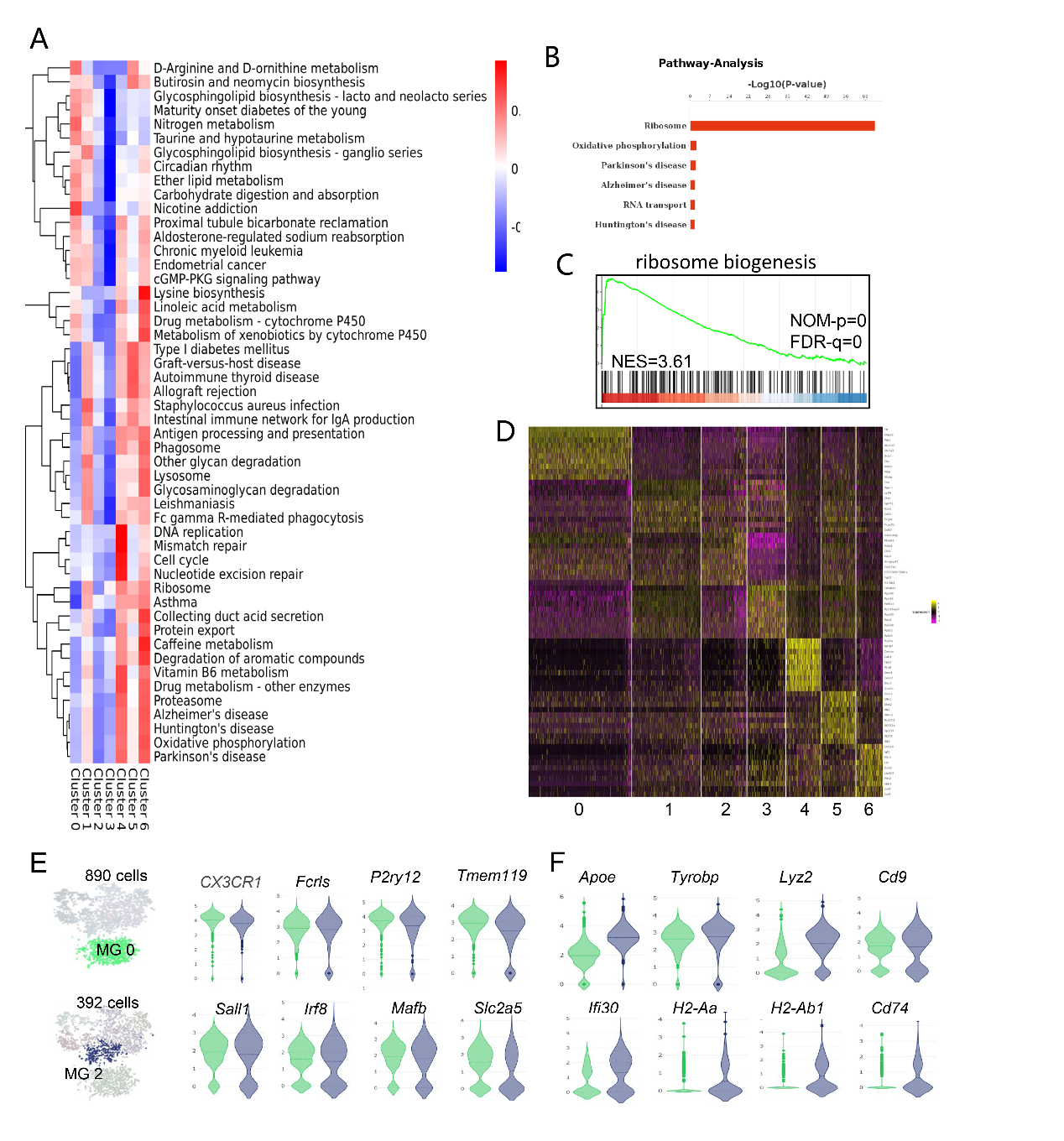
**

**Figure S3 Functional characterization of microglia subsets.**

(A) Heatmap reveals KEGG pathway enrichment of MG 0-6.

(B) GO biological process based on DEGs of MG 3.

(C) GSEA using DEGs of MG3 reveals positive enrichment of ribosome biogenesis, with normalized enrichment score (NES) = 3.61.

(D) Heatmap shows the top 10 expressed genes in MG 0-6. The color represents the scaled expression.

(E-F) Violin plots depict log-normalized expression of cluster-enriched genes of MG0&2.

**Figure S4**

**
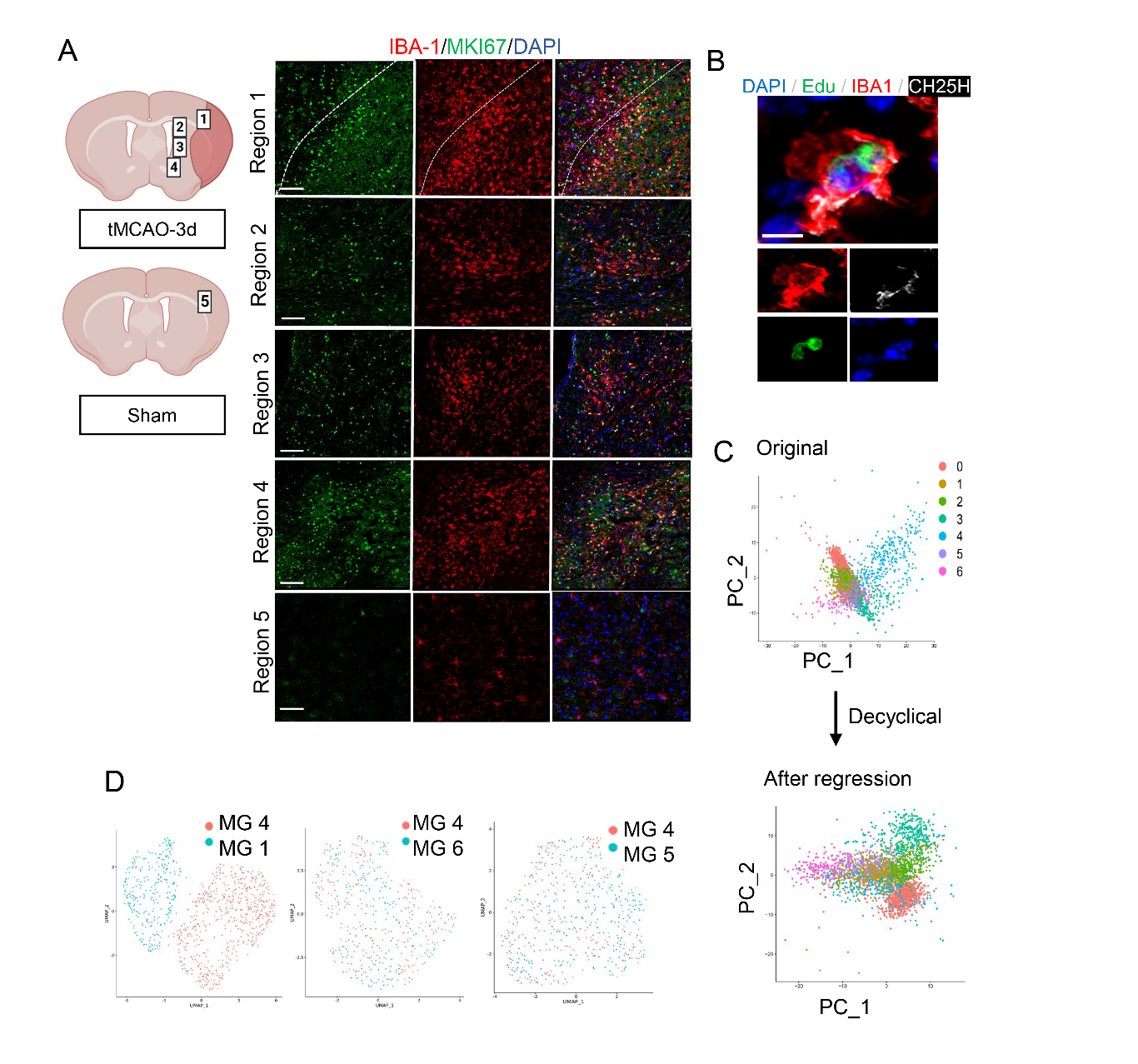
**

**Figure S4** **Representative immunofluorescence of proliferating microglia after ischemic stroke**

(A) Representative immunofluorescence pictures of post-3d-tMCAO mice showing the expression of Iba1and Mki67 on microglia in different CNS compartments. Scale bars, 100 μm (overview). Representative pictures of five mice from three independent experiments are depicted.

(B) Representative confocal images of CH25H, IBA-1 and EdU treble immunostaining of dividing cell in the ischemic penumbra 3d after tMCAO. Scale bar: 50 μm (overviews).

(C) Demonstrate of cell cycle regression in microglia.

(D) UMAP representation demonstrate MG4 (after cell cycle regression) with MG0, 5, 6, respectively.

**Figure S5**


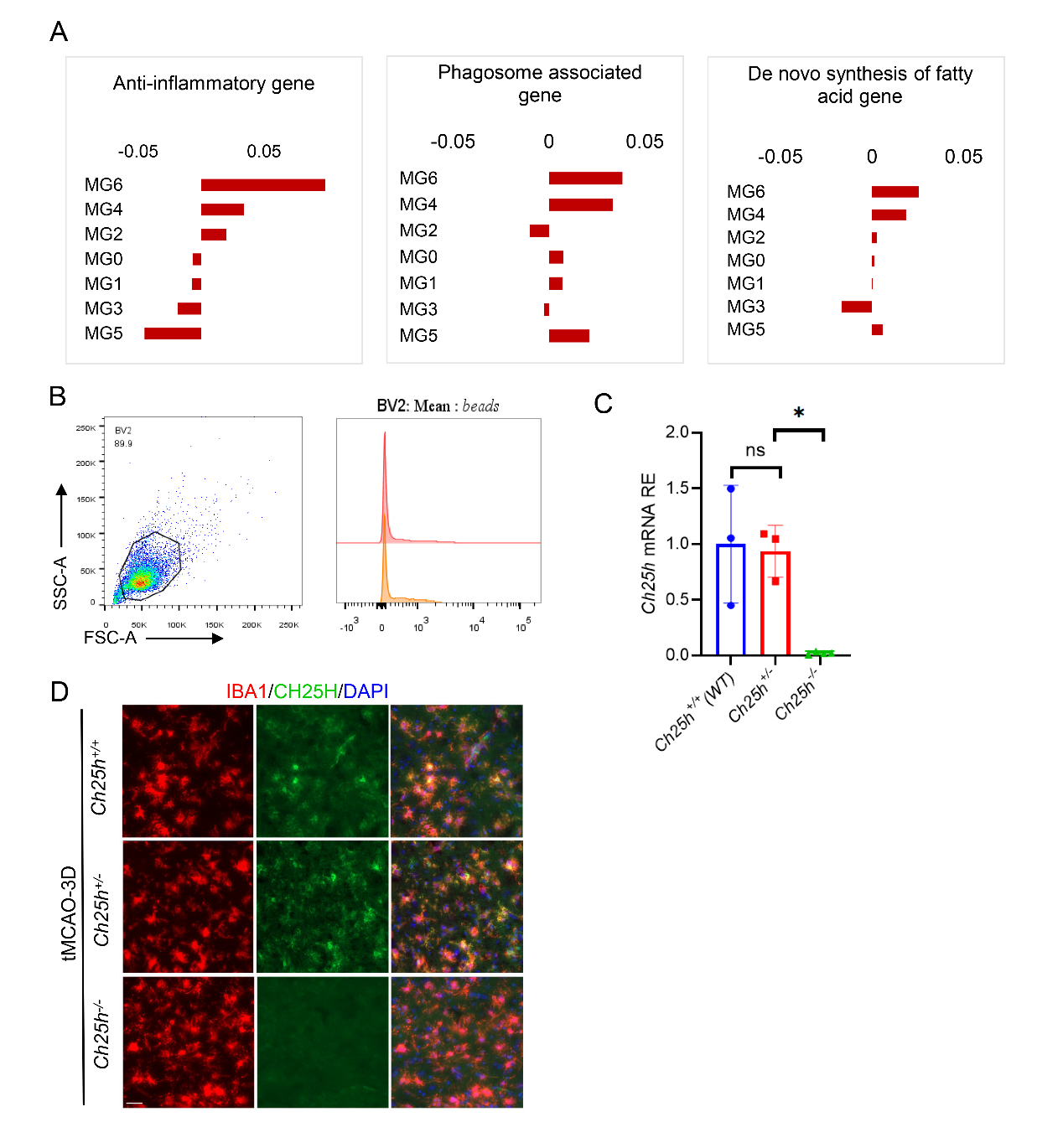


**Figure S5 *Ch25h* gene of the heterozygous was consistent with that of wild type mice**

(A)The score of anti-inflammatory, phagosome and de novo synthesis of fatty acid markers in different microglia clusters based on the analysis by QuSAGE (2.16.1).

(B) Representative dot plots and gating strategy for BV2 cells internalized fluorescent latex beads.

(C) qRT-PCR analysis of changes in the expression of Ch25h of post-3d-tMCAO ischemic brain from Ch25h^+/+^, Ch25h^+/-^and Ch25h^-/-^mice

(D) Representative immunofluorescence pictures of post-3d-tMCAO ischemic brain showing the expression of CH25H on microglia in Ch25h^+/+^, Ch25h^+/-^and Ch25h^-/-^mice

**Figure S6**

**
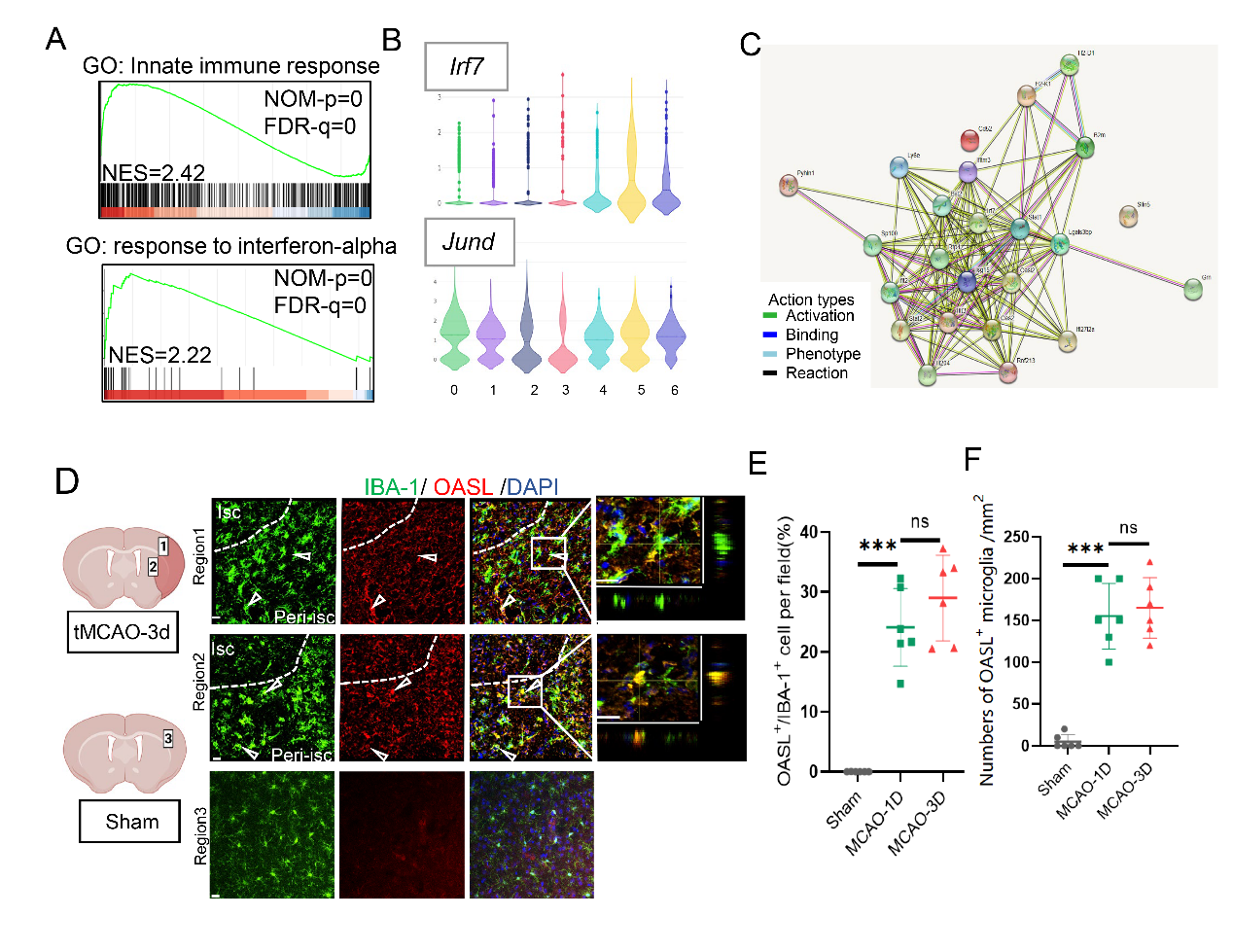
**

**Figure S6 Identification of an OASL^+^ microglia subset sensitive to type Ⅰ interferon in post-stroke mice brain**

(A) GSEA using DEGs of MG5 reveals positive enrichment of innate immune response and response to interferon-alpha.

(B) Violin plot of Irf7 and Junb in all microglia clusters.

(C) Interactome of top 20 specific genes in MG5.

(D) Confocal images of IBA-1, and OASL in brain sections obtained from post-3d-tMCAO mice brain (cortex and striatum). Scale bars, 20μm. Triangles indicate resident OASL^+^ microglia.

(E-F) Quantification OASL^+^ IBA-1^+^ cells in the brain. (n=5/6 per group, one-way ANOVA with Bonferroni multiple comparisons test). The data are shown as means ± SD. *p < 0.05, ***p < 0.001, n.s. no significance.

**Figure S7**


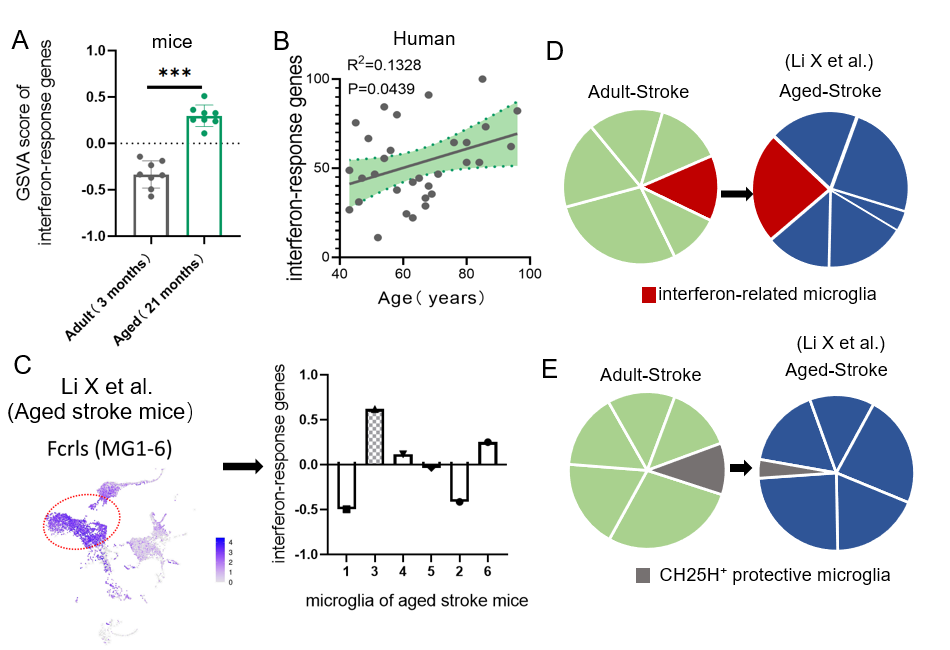


**Figure S7 The accumulation of neuroinflammatory OASL+ subcluster was increased in the aged ischemic mice brain.**

(A) GSVA score of the 60 most central genes in the interferon-response module is greater in microglia isolated from aged wild-type relative to young adult mice (6–8 weeks versus 16–18 months of age; n = 7 mice per group, GSE127893). Data are shown as mean ± SD. Student’s t-test; **p < 0.01.

(B) Linear regression of the mean normalized expression of the 60 most connected genes from the human interferon-response network in microglia isolated from the cortex of humans with different ages (34–102 years of age, GSE135437).

(C) GSVA score of interferon-response genes for each microglia cluster in aged stroke mice.

(D) The proportion of OASL^+^ microglia in adult brain and aged brain after ischemic stroke.

(E) The proportion of CH25H^+^ microglia in adult brain and aged brain after ischemic stroke.

(OASL, 2'-5'-oligoadenylate synthetase Like; GSVA, gene set variation analysis; IBA-1, ionized calcium binding adapter molecule 1; tMCAO, transient middle cerebral artery occlusion; dMCAO, distal middle cerebral artery occlusion.)

**References**

1. Cekanaviciute E, Fathali N, Doyle KP, Williams AM, Han J, Buckwalter MS. Astrocytic transforming growth factor-beta signaling reduces subacute neuroinflammation after stroke in mice. *Glia*. 2014;62:1227-1240

2. Rogers DC, Campbell CA, Stretton JL, Mackay KB. Correlation between motor impairment and infarct volume after permanent and transient middle cerebral artery occlusion in the rat. *Stroke*. 1997;28:2060-2065; discussion 2066

3. Shiotsuki H, Yoshimi K, Shimo Y, Funayama M, Takamatsu Y, Ikeda K, et al. A rotarod test for evaluation of motor skill learning. *J. Neurosci. Methods*. 2010;189:180-185

4. Vorhees CV, Williams MT. Morris water maze: Procedures for assessing spatial and related forms of learning and memory. *Nat. Protoc.* 2006;1:848-858
